# Development and In Vitro/In Vivo Comparative Characterization of Cryopreserved and Decellularized Tracheal Grafts

**DOI:** 10.3390/cells12060888

**Published:** 2023-03-13

**Authors:** Elena Stocco, Silvia Barbon, Marco Mammana, Diletta Trojan, Alice Bianchin, Francesca Favaretto, Martina Contran, Giovanni Zambello, Andrea Vogliardi, Marta Confalonieri, Silvia Todros, Piero G. Pavan, Filippo Romanato, Maria Teresa Conconi, Veronica Macchi, Raffaele De Caro, Federico Rea, Andrea Porzionato

**Affiliations:** 1 Section of Human Anatomy, Department of Neuroscience, University of Padova, 35121 Padova, Italy; 2 L.i.f.e.L.a.b. Program, Consorzio per la Ricerca Sanitaria (CORIS), Veneto Region, 35128 Padova, Italy; 3 Foundation for Biology and Regenerative Medicine, Tissue Engineering and Signaling-TES, Onlus, 35136 Padova, Italy; 4 Thoracic Surgery Division, Department of Cardiac, Thoracic, Vascular Sciences and Public Health, Padova University Hospital, Via Giustiniani, 2, 35128 Padova, Italy; 5 Tissue Bank, Fondazione Banca dei Tessuti del Veneto ETS, 31100 Treviso, Italy; 6 Department of Physics and Astronomy ‘G. Galilei’, University of Padova, 35131 Padova, Italy; 7 Department of Industrial Engineering, University of Padova, 35131 Padova, Italy; 8 Fondazione Istituto di Ricerca Pediatrica Città della Speranza, 35127 Padova, Italy; 9 Department of Pharmaceutical and Pharmacological Sciences, University of Padova, 35131 Padova, Italy

**Keywords:** trachea, decellularization, cryopreservation, tracheal cartilage, respiratory epithelium, immunogenicity, tissue engineering

## Abstract

Tracheal reconstruction represents a challenge when primary anastomosis is not feasible. Within this scenario, the study aim was to develop a new pig-derived decellularized trachea (DecellT) to be compared with the cryopreserved counterpart (CryoT) for a close predictive analysis. Tracheal segments underwent decellularization by a *physical* + *enzymatic + chemical* method (12 cycles); in parallel, cryopreserved samples were also prepared. Once decellularized (histology/DNA quantification), the two groups were characterized for Alpha-Gal epitopes/structural proteins (immunohistochemistry/histology/biochemical assays/second harmonic generation microscopy)/ultrastructure (Scanning Electron Microscopy (SEM))/mechanical behaviour. Cytotoxicity absence was assessed in vitro (extract-test assay/direct seeding, HM1SV40 cell line) while biocompatibility was verified in BALB/c mice, followed by histological/immunohistochemical analyses and SEM (14 days). Decellularization effectively removed Alpha-Gal epitopes; cartilage histoarchitecture was retained in both groups, showing chondrocytes only in the CryoT. Cryopreservation maintained few respiratory epithelium sparse cilia, not detectable in DecellT. Focusing on ECM, preserved structural/ultrastructural organization and collagen content were observed in the cartilage of both; conversely, the GAGs were significantly reduced in DecellT, as confirmed by mechanical study results. No cytotoxicity was highlighted by CryoT/DecellT in vitro, as they were also corroborated by a biocompatibility assay. Despite some limitations (cells presence/GAGs reduction), CryoT/DecellT are both appealing options, which warrant further investigation in comparative in vivo studies.

## 1. Introduction

Tracheal resection with primary end-to-end anastomosis (TRA) is the treatment of choice for several conditions leading to airway narrowing (e.g., trauma, infections, tumor growth, softening, and congenital tracheal stenosis). However, this therapeutic option is not applicable if the affected length exceeds 50% of the trachea in adults and 30% in children, being associated with high risk of suture tension and anastomosis failure [1,2].

To date, surgical management of patients suffering from severe tracheal lesions (mainly neoplastic), that are not eligible for TRA, still represents a significant challenge; endotracheal stents, tracheostomy tubes or Montgomery T-tubes are the only established treatment option in such clinical conditions, even though infection, stent migration, mucous plugging, or granulation tissue formation are common [1]. Within this scenario, intense efforts have been devoted towards the identification of an effective tracheal circumferential substitute. The requirements include lateral stiffness with longitudinal flexibility, adequate and airtight lumen, biocompatibility with low toxic/immunogenic effects, instructive behaviour for epithelial cell growth, and integration into adjacent tissues [3,4,5,6].

Graft characteristics directly affect the clinical outcome of the implant; hence, trying to address the “ideal substitute” needs, several synthetic prostheses (Dacron, polyurethane mesh, polytetrafluoroethylene, polypropylene mesh, silicone rubber, and even glass tubes [7]), autologous tissues composites (e.g., free periosteal, jejunal, muscular, oesophageal, bronchial and aortic grafts [8]), tracheal transplantation, allografts, and tissue engineering-based devices have been attempted for trachea reconstruction over the years. Unfortunately, none distinguished among the others for fully satisfactory outcomes in vivo [9,10,11]. Scar tissue formation with graft stenosis and obstruction, not adequate biomechanical characteristics, required stenting to avoid collapse, lifelong immunosuppressive therapies, and technical surgical challenges were included among the main critical issues [12]. Additionally, as supported by preclinical and early clinical evidence, incomplete or even absent re-epithelization of the graft was possibly encountered, responsible for impaired mucus clearance, recurrent infections, and consequent implant failure [13,14]. Despite the need for further investigation, two alternatives may be promising due to a specific anatomical origin and possibly reduced/absent immunogenicity, which also makes them suitable for cancer patients who cannot tolerate immunosuppressive therapies [15]. These include the cryopreserved tracheal allografts and the decellularized tracheal substitutes.

Cryopreservation is one of the most common procedures for allografts storage. After freezing by a thermally controlled procedure in the presence of a cryoprotectant, the tissue is preserved in vapor phase liquid nitrogen for a determined period (according to validations or directives), prior to being thawed under controlled warming conditions before use [7]. Several cryopreserved tissues are currently available in clinical practice including, for instance, amniotic membrane [16], bone [17], menisci [18], vascular tissues [19,20], and heart valves [21,22]. Cryopreserved tracheas show a native-like tubular structure, maintaining histological characteristics and mechanical properties and possibly reducing immunogenicity. This event occurs as a consequence of class II leukocyte antigen expression depletion, due to tracheal epithelium exfoliation during the freezing and thawing [23,24,25]. There is consensus in identifying the respiratory epithelium and lamina propria as the main targets for rejection; thus, cryopreservation, altering their integrity, may be a strategy of interest triggering a decline in allograft antigenicity. However, the overall procedure-related effects are still disputed due to conflicting evidence ascribable to preserved tracheal cartilage viability (chondrocytes are immunoprivileged) as well as epithelium and lamina propria partial maintenance [8,25,26,27]. According to our knowledge, despite sounding promising, the use of cryopreserved (−70 °C) (and irradiated) tracheal homograft in humans was only reported by Kunachak et al. [28] for cervical laryngotracheal reconstruction in four patients (follow-up: 18–20 months). No significant evidence of immunologic rejection was observed, even though a larger series and a longer follow-up time are required.

Decellularization is a common method for preparing tissue-engineered tracheal scaffolds that ideally retain mechanical properties, lumen patency, and support epithelial cell growth and angiogenesis, without triggering an immunogenic response thanks to the cellular components removal [25,29,30]. A wide variety of approaches are available based on physical, enzymatic, and chemical strategies. Briefly: physical treatments or ionic solutions induce breaking of the cell membrane; enzymatic treatments separate cellular components from the ECM; detergents solubilize the cytoplasmic components. Intensive washes with deionized water are also adopted to remove cellular debris [11]. Currently, a great debate exists over the “decellularization grade”: completely acellular supports and partially decellularized supports (removal of immunogenic cells and immunoprivileged chondrocytes preservation) are both under consideration [2,31,32,33]. A higher cells’ removal is associated with a lower risk in immune/inflammatory responses; however, maintenance of the structural/ultrastructural features and density of the target tissue are also key elements of an efficient decellularization protocol [34]. To date, clinical transplantation of a decellularized and bioengineered tracheal allograft is a procedure still considered as compassionate [35,36].

Despite both being appealing, consensus over the optimal strategy is still lacking [37]. Several knowledge gaps exist about the effects of cryopreservation and thawing on tracheal tissue cell viability, immunogenicity, and extracellular matrix features [38]; furthermore, recurring to decellularization methods gives rise to several questions mainly related to ECM features and ultrastructural modification descending from the impact of potential “aggressive” protocols towards ultrastructure integrity. Hence, the aim of this study was to develop and describe pig-derived tracheal grafts obtained by cryopreservation and decellularization for a close comparison over tissue structural and ultrastructural features, ECM characteristics, eventual cytotoxicity descending from the preparation protocols, and biocompatibility in vivo. The results can be predictive for device outcomes after orthotopic implantation.

## 2. Materials and Methods

### 2.1. Tracheal Isolation

Donor tracheae were harvested from adult pigs (*n* = 10) weighing approximately 130 kg. The animals were sacrificed and the entire airway from the larynx to the lungs was extracted en bloc with the oesophagus. Then, two tracheal segments per animal, of approximatively 5 cm in length, were carefully isolated, cleaned from mucus, and put into antibiotic solution composed of BASE medium (Alchimia S.r.l., Ponte San Nicolò, Padova, Italy), gentamicin 200 µg/mL (Fisiopharma, Palomonte, Salerno, Italy), vancomycin 100 µg/mL (Fisiopharma) and meropenem 200 µg/mL (Venus Pharma, Werne, Germany) at +2 °C/+8 °C.

The two segments were intended for cryopreservation and decellularization, respectively; however, as for control, samples of 1 cm in length/animal were excised from tracts assigned to decellularization and properly fixed for subsequent comparative analyses.

### 2.2. Tracheal Cryopreservation

Cryopreservation of the tracheal segments (*n* = 10) was carried out by the tissue bank “Fondazione Banca dei Tessuti del Veneto ONLUS” (FBTV), in accordance with the requirements approved by the National Transplant Centre and following a method previously described [39]. Briefly, after the first overnight decontamination, the segments were again cleaned from mucus, valuated in their integrity, and further decontaminated with the same antibiotic cocktail composed of BASE medium (Alchimia S.r.l.), gentamicin (Fisiopharma), vancomycin (Fisiopharma), and meropenem (Venus Pharma) at +2 °C/+4 °C for at least 48 h [40,41]. At the end of the second decontamination step, tissues were immersed and washed in sterile saline solution for at least 5 min (min). Samples of the washing solutions were sent to Istituto Zooprofilattico Sperimentale delle Venezie to verify the absence of contaminants. Hence, before cryopreservation, the tracheas were placed within low temperature-resistant ethylene-vinyl acetate bags with a solution composed of BASE medium (Alchimia S.r.l.), 10% dimethylsulfoxide (DMSO) (Wak-Chemie Medical GmbH, Steinbach (Taunus), Germany), and 10% human serum albumin (Alburex 20%, CSL Behring GmbH, Milano, Italy). Cryopreservation was performed by means of a programmable cryogenic freezer (Planer KryoSave Integra, 750-30; Planer Limited, Middlesex, UK), which triggered a controlled cooling rate up to −140 °C. Tissues were stored at temperatures below −160 °C in liquid nitrogen vapor phase and thawed before use [42,43].

All procedures were carried out under a laminar flow hood; sterile disposable materials and solutions were used to avoid tissue contamination.

### 2.3. Tracheal Decellularization

The tracheal segments were decellularized according to a partially modified Tergitol^TM^-based method, previously described by Barbon et al. [44,45]. Briefly, the samples (*n* = 10) were preliminarily decontaminated through 3 washes of 30 min each in a 2% P/S solution in sterile dH_2_O (+4 °C, under stirring). Hence, they underwent a physical + enzymatic + chemical treatment protocol providing for: (i) tissues freezing (−20 °C/12 h) and lyophilization (12 h) (FreeZone 2.5 Liter Benchtop Freeze Dryer, Labconco™, Kansas City, MO, USA); (ii) enzymatic treatment with Deoxyribonuclease I (10,000 Kunitz units of DNase-I from bovine pancreas-5VL, Sigma Aldrich, St. Louis, MI, USA) in 1M sodium chloride (NaCl) solution (room temperature (RT)/6 h); washing with dH_2_O and treatment with 0.05% trypsin + 0.02% EDTA enzymatic solution in dH_2_O (37 °C/1 h), followed by extensive rinsing in dH_2_O; (iii) washing with 2% Tergitol^TM^ detergent solution + 0.8% ammonium hydroxide (NH_4_OH) in dH_2_O (+4 °C/3 days, under stirring); (iv) washing with dH_2_O (+4 °C/2 days, under stirring) (1 cycle included the phases i–iv).

After phase (iv), the samples were frozen at −20 °C prior to being lyophilized as in (i). To obtain acellular tracheal segments, the decellularization was repeated for 12 cycles. At the end of the decellularization, the lyophilized segments were stored at −20 °C until their use.

Each phase involving tissue manipulation was performed under a second level biological hood; sterile filtered/autoclaved solutions and sterile disposable material were used with the aim of preventing any contamination.

### 2.4. 4′,6-Diamidino-2-Phenylindole Nuclear Staining

Cryopreserved and decellularized tracheal tissues were compared to native trachea by a fluorescence analysis based on 4′,6-Diamidino-2-Phenylindole (DAPI) aiming to assess nuclei presence/distribution. The epithelial layer, the mucosa, and submucosa layers and the cartilaginous compartments were all considered, for a full-thickness description of the tissue. Briefly, small tissue samples were excised, Optimal Cutting Temperature (OCT) medium embedded and frozen prior to being cut into 5 µm thick sections using a cryomicrotome (Leica CM 1850 UV; Leica Microsystems, Wetzlar, Germany); hence, the sections were fixed with acetone, mounted with Vectashield mounting medium for fluorescence with DAPI (Vector Laboratories, Burlingame, CA, USA) and photomicrographs were acquired with a Leica LMD6 (Leica Microsystems) connected to a Leica DFC320 high-resolution digital camera (Leica Microsystems) and a computer equipped with software for image acquisition (LasX, Leica Microsystems).

### 2.5. DNA Extraction and Quantification

Together with DAPI, DNA quantification was also performed to verify in which extent the immunogenic material was detectable in cryopreserved and decellularized tracheas, versus native tissue; to this purpose, the DNeasy Blood and Tissue Kit (Qiagen, Düsseldorf, Germany) was used. Briefly, following the manufacturer’s protocol, a certain weighted amount of tissue (10 mg) was Proteinase K (Merck Life Science, Darmstadt, Germany)-lysed at 56 °C, overnight. Hence, the lysates were loaded onto the DNeasy Mini spin columns, allowing for a total DNA selective purification, and the eluted DNA was finally quantified according to a fluorometric method using a Qubit 4 fluorometer and kit (ThermoFisher Scientific, Waltham, MA, USA). Three replicates/group were considered.

### 2.6. Alpha-Gal Epitope Detection

Superficial epitopes, such as Alpha-Gal, may trigger hyperacute and acute vascular rejection phenomena after xenograft positioning [46]. Hence, immunohistochemistry was adopted to verify if tissue treatments were able to mask or inactivate the epitope Alpha-Gal.

Tracheal samples were fixed in 10% formalin solution, paraffin-embedded and then cut into 3 µm thick sections. After dewaxing with a series of ethanol (Arco Scientifica S.r.l., Padova, Italy) solutions (99%, 95%, and 70%) and rehydrating in distilled water, immunohistochemical reaction was performed by Dako Autostainer/Autostainer Plus (Dako, Milan, Italy) with the anti-Alpha-Gal epitope primary antibody (monoclonal mouse anti-rabbit, LS-C63415, LSBio, Seattle, WA, USA). The binding between primary antibody and specific antigen was then revealed using a labeled polymer (EnVision™ FLEX-HRP; Dako) and 3,3′-diaminobenzidine (EnVision™ FLEX Substrate buffer + DAB + Chromogen; Dako). In the meanwhile, negative controls were developed without incubation with primary antibodies.

### 2.7. Histological Analyses

Formalin-fixed and paraffin-embedded sections (5 µm thickness) were dewaxed and rehydrated with a series of ethanol (Vetrotecnica, Padova, Italy) solutions (99%, 95%, 70%) before being stained according to laboratory routine protocols.

Haematoxylin and eosin (H&E) staining was adopted to verify cells nuclei presence/distribution (hematoxylin, deep blue-purple color) and ECM integrity (eosin, pink staining). Dewaxed and rehydrated tissue slices were immersed in hematoxylin for 1 min (Sigma-Aldrich, St. Louis, MO, USA), tap water for 5 min, and eosin (Sigma-Aldrich) for 1 min. The sections were then dehydrated in a series of ethanol (95% for 45 s (s) and 100% for 3 min (×2)), and finally immersed in xylene. Each section was then mounted with Eukitt (Merck LifeScience, Bayswater VIC, Australia).

Masson’s Trichrome and Sirius Red staining allowed to evaluate the connective tissue, typically collagen fibers. Briefly, Masson’s Trichrome staining was performed using the Masson trichrome staining kit (Bio-Optica, Milano, Italy), according to the manufacturer’s instructions. The rehydrated sections were exposed to Weigert’s iron haematoxylin (A solution) + Weigert’s iron haematoxylin (B solution) (for 10 min); without washing, the slides were drained, and picric acid alcoholic stable solution (C solution) was poured on the sections and allowed to act (for 4 min). Thus, after a quick wash in distilled water (3–4 s), Ponceau acid fuchsin according to Masson (D solution) was added to the section (for 4 min). Following a wash in distilled water, phosphomolybdic acid solution was added to the section (E solution) (for 10 min). Without washing, the slides were drained and a light green solution according to Goldner (reagent F) was left to act (for 5 min). Finally, the sections were washed in distilled water, dehydrated, and cleared in xylene as previously described and mounted with Eukitt (Merck LifeScience). Regarding Sirius Red, rehydrated sections were immersed in Picrosirius Red solution (Sigma-Aldrich) for 30 min, washed in deionized acidified water (HCl, 0.1%), washed in deionized water and then dehydrated and cleared in xylene (like above) before mounting with Eukitt (Merck LifeScience).

Alcian-blue staining was conducted to assess the acidic mucins and the GAGs retained in the submucosa and in the cartilage/adventitia, respectively. According to the staining protocol, rehydrated sections were exposed to Alcian-blue solution (Sigma-Aldrich) for 30 min. Hence, the slices were washed with tap water for 2 min, counterstained with hematoxylin (Sigma-Aldrich) for cells nuclei detection, washed with tap water for 5 min, dehydrated and cleared in xylene prior to be mounted with Eukitt (Merck LifeScience).

Weigert Van Gieson staining aimed at demonstrating the preservation of elastic fibres; to this purpose, the Weigert Staining Kit for Elastic Fibers-Rapid Method (Bio-Optica) was used. Following the manufacturer’s instructions, the rehydrated slices were exposed to periodic acid solution (A solution) for 5 min; after rinsing the samples in distilled water, an incubation box was prepared in which the alcoholic reagent for incubation (B solution) and the slides were dropped, covered with Weigert’s resorcin fuchsin (C solution), were incubated into for 30 min; thereafter, the sections were rinsed in distilled water and exposed to the acid differentiation buffer (D solution) for 2 min. Once washed in tap water (5 min) and rinsed in distilled water, carmalum according to Mayer (E solution) was put on the sections for 5 min before rinsing in distilled water. Dehydration, clearing in xylene and mounting with Eukitt (Merck LifeScience) occurred as previously described.

#### 2.7.1. Morphometric Analyses

Semi-quantitative analysis of ECM elements in native, cryopreserved, and decellularized tracheas was performed by previously standardized protocols [45,47]. Photomicrographs were acquired under a Leica DM4500B microscope (Leica Microsystems) connected to a Leica DFC320 high-resolution digital camera (Leica Microsystems). An ImageJ software was used for quantifications as described below.

Semi-automatic immunoreactivity quantification (Alpha-Gal Epitopes) was calculated on an average of three ×20 magnification photomicrographs/section as previously established [48,49].

Collagen and elastic fibers were quantified as percentage areas stained in green (collagen) and purplish (elastin) with Masson’s Trichrome and Weigert Van Gieson, respectively. Images of stained sections were acquired in bright field at a ×5 and ×10 magnification. Green and purplish areas were identified by displaying histograms of the distribution of hue, saturation and brightness and setting adequate thresholds for each of these parameters, which were maintained for all the morphometric analyses. Specific hue, saturation and brightness ranges were, respectively, 50–138, 0–255, and 0–185 for green colour (collagen) and 127–211, 0–255, and 0–185 for purplish colour (elastic fibers). The coloured area corresponding to collagen/elastic fibres were selected and automatically measured and results were finally presented as percentage areas stained in green/purplish out of the total area of the acquisition field.

At the same time, type I and III collagen content was quantified on Sirius red-stained sections. After acquiring images by polarized microscopy at ×5 magnification and saving them as TIFF files, red-orange and green-yellow areas were identified by setting adequate thresholds of the hue component. In particular, hue, saturation and brightness ranges were, respectively, 0–39, 189–255, and 60–203 for red-orange, 210–255, 78–255, and 60–150 for red and 39–120, 78–255, and 135–255 for green-yellow. Mean percentage red-orange and green-yellow areas out of the total area of the image were finally calculated to define collagen content in native, cryopreserved and decellularized tracheas.

### 2.8. Biochemical Assay for Glycosaminoglycans Quantification

The sulphated glycosaminoglycans (GAGs) were quantified through the Chondrex Inc. Glycosaminoglycans Assay Kit (DBA Italia S.r.l., Milan, Italy), following the Manufacturer instructions. Briefly, a weighted amount of tissue samples (10 mg) was preliminarily digested in Papain solution at 56 °C, overnight to induce GASs solubilization; hence, the solubilized GAGs were labeled by the cationic dye 1,9 dimethylmethylene blue (DMB) and the colorimetric reaction was read at 530 nm using a Microplate auto reader VICTOR3™ (PerkinElmer, Waltham, MA, USA).

Together with cryopreserved and decellularized trachea samples, chondroitin sulphate standard was also analysed to allow for GAGs quantification into the specimens.

### 2.9. Scanning Electron Microscopy

Tracheal tissues were analysed for their ultrastructure considering both the external and the luminal sides. The samples were fixed with 2.5% glutaraldehyde in 0.2 M phosphate buffer solution (PBS) (pH 7.4) for 24 h, washed 5 times in PBS to remove chemical residues and then dehydrated with a graded ethanol series. After critical point drying and gold sputtering, micrograph acquisition was performed by using the tungsten thermionic emission SEM system JSM-6490 (Jeol USA, Peabody, MA, USA).

### 2.10. Two Photon Microscopy

Second Harmonic Generation (SHG) imaging was performed through a custom developed multiphoton microscope [50]. Briefly, an incident wavelength of 800 nm (~40 mW average laser power, under the microscope objective) was adopted to detect the collagen’s SHG signal at 400 nm. The images were acquired at a fixed magnification through the Olympus 25× water immersion objective with 1.05 numerical aperture (1024 × 1024 pixels), averaged over 100 consecutive frames, with a pixel dwell time of 0.14 μs and a pixel width of 0.8 μm. Coherency (C) was determined to assess the local dominant orientation of collagen fibers; to achieve that, it was used the ImageJ plugin OrientationJ, [51]. The estimated parameter is bounded between 0 and 1, indicating, respectively, the absence (isotropy) and the presence (anisotropy) of the dominant orientation. A graphic representation of the coherency, showing organization and distribution of the fibres, is achieved by Fast Fourier Transform (FFT) analysis. The transform-based texture analysis techniques convert the image into a new form using the spatial frequency properties of the pixel intensity variations allowing the extraction of textural characteristics from the image. Highly oriented fibre in a single direction shows an elliptic shape; whether a circular shape represents fibre spread in all directions [44,45,47,50,52,53]. Five samples/group were considered in this analysis; for each sample, 5 different areas were analyzed.

### 2.11. Compressive Mechanical Tests

**Figure 1:**
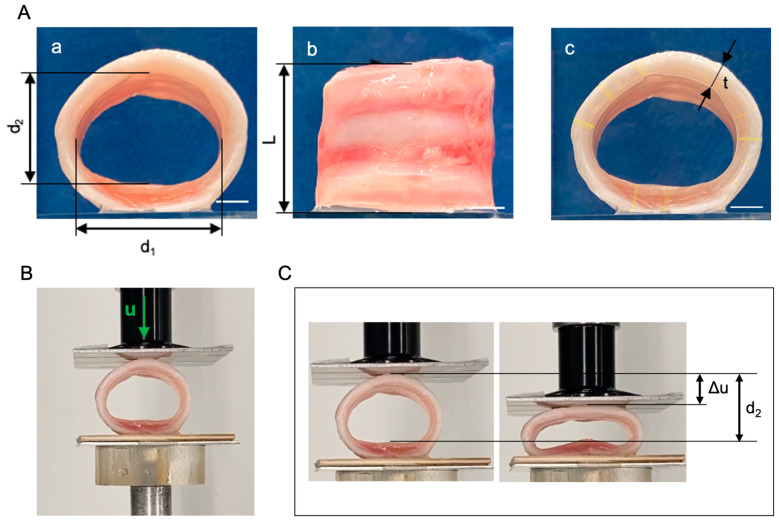
(**A**) Ring-like sample of trachea for mechanical testing, with measurements of the medio-lateral (d_1_) and proximal-distal (d_2_) diameters (**a**), the length of the segment L (**b**) and the tracheal wall thickness t (**c**). Yellow segments refer to the shortest distance between the inner and outer outline of the sample section in 30 random points. Scale bars = 5 mm. (**B**) Experimental set-up for compression tests with trachea sample between two flat plates. The displacement Δu of the upper plate is applied along the distal-proximal direction. (**C**) Undeformed and deformed trachea samples with reference to the plate displacement Δu and the initial diameter d_2_.

Segments of trachea from three donors (respectively, NativeT, DecellT and CryoT) were cut transversally into ring-like samples including three cartilaginous structures. At least three samples were obtained for each trachea. The shape and size of each sample were evaluated by measuring the medio-lateral and proximal-distal diameters (d_1_ and d_2_, respectively), the tracheal wall thickness t and the length of the segment L (Figure 1A), by means of the image analysis software Fiji [51]. Specifically, the mean thickness of each sample was obtained by measuring in the transversal view the shortest distance between the user-drawn inner lumen and outer outline in 30 randomly selected points (Figure 1B). These data were used to calculate the average wall thickness.

Compression tests were carried out with Bose ElectroForce^®^ Planar Biaxial Test Bench instrument (TA Instruments, New Castle, DE, USA) under displacement control, with a load cell of 22 N. Two flat plates were positioned parallel to each other (Figure 1C): samples were placed on the lower plate, leaning on the posterior tracheal wall, while the upper plate was gradually approached at a constant rate of 0.2 mm s^−1^. Samples were compressed up to reducing of 50% the initial proximal-distal diameter. Tracheal compressive deformation *s* in the proximal–distal direction was measured as the ratio of the plate displacement Δu and the initial diameter d_2_ (Figure 1D). To compare tracheal samples of different sizes, the force per unit of length of the sample f (in N mm^−1^) was calculated as the ratio between the force value F measured by the load cell and the sample length L. The stiffness of the sample was estimated by considering the secant compressive modulus k, calculated as the slope of the straight line drawn from the experimental data at 20% and 50% compressive deformation. The values of the secant compressive modulus k were analyzed through Kruskal–Wallis nonparametric one-way Analysis of Variance (ANOVA) and post hoc comparison, considering as significant a *p*-value lower than 0.05.

### 2.12. Tissue Cytotoxicity and Biocompatibility

Tracheal tissue cytotoxicity and biocompatibility were assessed in vitro and in vivo, respectively.

Differently from the cryopreserved samples, acellular tissues were sterilized before the assays to exclude eventual contamination occurring during the decellularization process, as it implied extensive manipulation of the tissues. Briefly, the samples underwent three washes of 30 min each, in a 2% P/S solution in sterile dH_2_O (+4 °C, under stirring), followed by exposure to UV light for 30 min/side.

#### 2.12.1. In Vitro Cytotoxicity

3(4,5-dimethylthiazole-2-yl)-2,5-diphenyltetrazolium-bromide (MTT) (Merck Life Science) assay was performed to exclude cytotoxicity of cryopreserved and decellularized matrices, ascribable to chemical remnants adsorption and release. Considering the potential of mesenchymal stem cells (MSCs) for trachea regenerative purposes [54], human bone marrow-derived stromal cells (HM1-SV40, immortalized cell line) were used within this study.

Preliminarily, to obtain the tissue extracts, a weighted amount (400 mg) of decontaminated cryopreserved and decellularized tracheal samples were incubated in the HM1-SV40 cell proliferation medium (1 mL of medium/100 mg of tissue) consisting in Alpha-Modified Eagle Medium (α-MEM) (ThermoFisher Scientific), 16.5% of fetal bovine serum (FBS) (ThermoFisher Scientific), 1% glutamine (Merck Lifescience) and 1% penicillin/streptomycin solution (100 mg/mL) (Merck Lifescience). Incubation lasted 72 h at 37 °C (5% CO_2_ and 95% humidity).

In parallel, 20,000 HM1-SV40 cells/well were seeded on 24-well culture plates (Corning). After 24 h, the cell culture medium was removed and replaced with the extract medium. As positive (cytotoxic) control, culture medium added with 50% dimethyl sulfoxide (DMSO; Sigma-Aldrich) was used, whereas the negative control was represented by untreated cultures. Both treated and controlled cultures were maintained for 24 h at 37 °C, 95% relative humidity and 5% CO_2_. The effect of extract medium on cell survival was then evaluated by the MTT assay.

At the scheduled end-point, the cells were at first observed at the optical microscope and the culture medium was replaced with 0.5 mg/mL MTT in α-MEM for 4 h; hence, the formazan precipitates were dissolved by 2-propanol acid (0.04 M HCl in 2-propanol) and optical density of the solutions was measured at 570 nm with the Microplate Auto Reader VICTOR3™ (PerkinElmer). Results of the cytotoxicity test were expressed as percentages of viable and metabolically active cells in treated groups versus the untreated control, which was set as 100% cell viability. It was possible to infer the number of cells through an MTT standard curve, obtained as previously described [45].

#### 2.12.2. Cell–Scaffold Interaction

Cytocompatibility of tracheal scaffolds was assessed also by investigating their ability to sustain the adhesion and proliferation of the immortalized human bone marrow cell line HM1-SV40. Cells were first expanded in culture by using proliferation medium (Section 2.12.1) until a sufficient number of cells was obtained for seeding experiments.

To prepare scaffolds for cell seeding, cryopreserved and decellularized tracheal patches (0.5 × 0.5 cm^2^) were sterilized by immersion in 2% antibiotic/antimycotic solution (Merck Lifescience) for 4 days under mild agitation, followed by extensive washes in PBS for 2 days under mild agitation and final incubation under UV light for 1 h. After maintaining tracheal scaffolds in cell proliferation medium overnight at 37 °C, 100,000 HM1-SV40 cells/support were seeded on the luminal side and cultured for 3 and 7 days before assessing cell growth on samples.

At the defined endpoints, SEM analysis was performed as described in Paragraph 2.9 to verify cell adhesion and proliferation on scaffold surface.

#### 2.12.3. In Vivo Biocompatibility

In vivo biocompatibility assay was performed implanting pig CryoT and DecellT samples in a subcutaneous pouch of Balb/C mice (International Organization for Standardization (ISO) 996-3) [55]. Animal surgery and husbandry were performed in accordance with the Italian guidelines on the use of experimental animals (DL n. 16/92 art. 5) and approved by the Ethical Committee of the University of Padova and by the Italian Department of Health (Authorisation n. 1076/2020-PR, 10 November 2020).

- Surgery

For in vivo biocompatibility study, disk-like tracheal samples were prepared from cryopreserved and decellularized tracheal matrices using a biopsy punch with 8 mm diameter. Hence, six twelve-week-old female mice were anesthetized using a binary gas mixture of isoflurane/oxygen and randomly assigned to the two experimental groups. After shaving and disinfecting the dorsal cutis with Betadine^®^ (Bayer, Leverkusen, Germany) a No. 10 surgical blade (Becton-Dickinson, Franklin Lakes, NJ, USA) was used to create a dorsal subcutaneous pouch of about 10 mm. Thereafter, the scaffolds were anchored to the *latissimus* dorsi muscle by using Tycron 4/0 sutures; the respiratory-epithelium side was put in direct contact with the muscular side. Finally, absorbable Novosyn 4/0 sutures were used to stich the skin. An adequate antibiotic/anti-inflammatory therapy was administered for 5 days after surgery. Euthanasia was performed after 14 days; hence, the scaffolds were excised with the surrounding tissues and properly fixed for subsequent histological and immunohistochemical analyses and SEM ultrastructural characterization, as previously described.

- Explants characterization

Histological analyses including H&E, Masson’s Trichrome, Sirius Red, Alcian-blue and Weigert Van Gieson staining were accomplished as previously reported to verify eventual ECM remodeling after in vivo implant. In addition, von Kossa staining was also included to establish a possible contribution of cryopreservation and decellularization to calcification of the specimens after implant. Briefly, re-hydrated sections were flooded with 5% of aqueous silver nitrate; hence they were exposed to sunlight for 20 min prior to be washed well in distilled water. The samples were than treated with 2% sodium thiosulphate for 2 min, washed in running tap water, rinsed in distilled water and counterstained with 1% Neutral red for 2 min. After a rapid dehydration, the slides were cleared in xylene prior to be mounted with Eukitt.

In parallel, immunological characterization for lymphomonocytic-fraction detection was performed with the following antibodies diluted in PBS: anti-CD3 (polyclonal rabbit anti-human CD3, A 0452; Dako, Milan, Italy) diluted 1:500 and anti-F4/80 (polyclonal rabbit anti-mouse anti-F4/80, sc-26643-R; Santa Cruz Biotechnology, CA, USA) diluted 1:800, to label lymphocytes and monocytes/macrophages, respectively. Antigen unmasking was performed with 10 mM sodium citrate buffer, pH 6.0, at 90 °C for 10 min. The sections were then incubated for 30 min in blocking serum [0.04% bovine serum albumin (BSA; A2153, Sigma-Aldrich) and 0.5% normal goat serum (X0907, Dako)] to eliminate unspecific binding, and then incubated for 1 h at RT with the above primary antibodies. Primary antibody binding was revealed by incubation with anti-rabbit/mouse serum diluted 1:100 in blocking serum for 30 min at RT (Dako^®^ EnVision^TM^ + peroxidase, rabbit/mouse; Dako, Glostrup, Denmark) and developed in 3,3′-diaminobenzidine for 3 min at RT. Lastly, the sections were counterstained with haematoxylin. As a negative control, sections were incubated without primary antibodies. Immunopositive elements were quantified as described in Section 2.7.1.

SEM analyses, conducted as described above, supported histological/immunohistochemical characterization for description of explants surfaces ultrastructure.

### 2.13. Statistical Analysis

Data are presented as mean ± standard deviation (SD) of at least three replicates. The one-way analysis of variance (ANOVA) followed by the Tukey post hoc test for multiple comparisons were used to determine any significant differences among the experimental groups. Unpaired *t*-test was used when comparing two groups. Differences were considered significant with *p* ≤ 0.05.

## 3. Results

### 3.1. Gross Appearance, Nuclear Staining, and DNA Content

Cryopreservation led to samples that after thawing (13 months of freezing) were characterized by an external whitish appearance and an internal pinkish lumen. The CryoT also maintained the tubular shape preserving lumen rigidity without evident differences versus NativeT.

**Figure 2:**
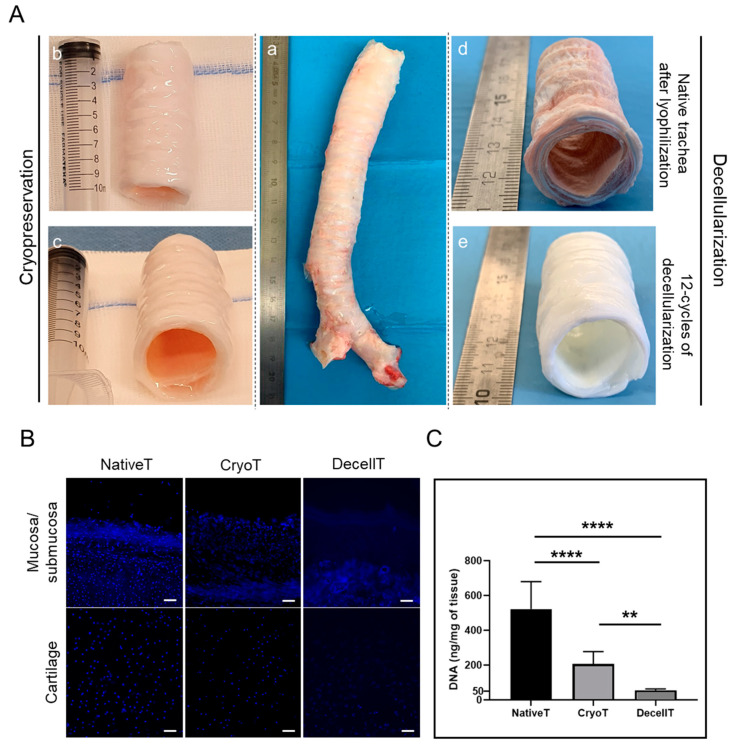
(**A**) Cryopreserved and decellularized pig trachea gross appearance. Soon after isolation (**a**) pig tracheas were divided into segments which underwent cryopreservation (**b**,**c**) and decellularization (**d**,**e**). Gross appearance of cryopreserved trachea segment (**b**,**c**); gross appearance of native tracheal segment after lyophilization (**d**); gross appearance of pig trachea after 12 decellularization cycles (**e**). (**B**) DAPI staining representative image of native (NativeT), cryopreserved (CryoT) and decellularized (DecellT) pig trachea. Blue dots (not detected in DecellT) correspond to cell nuclei. The presence of a diffused blue staining in the DecellT refers to extracellular matrix autofluorescence; autofluorescence was also detected in the Native T and CryoT samples. Scale bars = 100 μm. (**C**) Quantification of residual DNA into CryoT and DecellT samples versus the NativeT group (**: *p* < 0.01; ****: *p* < 0.0001).

Decellularization was accomplished in 12 weeks (12 decellularization cycles). Each cycle was introduced by a lyophilization treatment that imparted a wrinkled appearance to the graft but was not associated with evident tissue fractures/alterations. In addition, it guaranteed an optimal DNase-I solution penetration that is essential for protocol efficiency. Once rehydrated, the lyophilized samples returned to show a typical native tissue-like appearance. The decellularization protocol provided for segments with a white colour (both externally and internally), keeping a patent lumen without signs of collapse (Figure 2A).

DAPI staining allowed to detect presence/distribution of cellular nuclei and/or remnants within the CryoT and DecellT versus the native tissue, thus highlighting the protocol’s effectiveness in reducing the immunogenic potential of the samples. Typically, the native tissue displayed a broad and homogeneous distribution of the cells (nuclei, blue dots) in the whole tissue thickness (mucosa and submucosa, cartilage); in the CryoT group, the fluorescent elements were still identifiable, but they were reduced in number. DAPI staining results showed that only the DecellT group was mainly cell-nuclei free; however, despite blue dots being nearly absent, fluorescent remnants were detected, mainly located at the lacunae edges. A certain autofluorescence of the ECM was identified in all the experimental groups (Figure 2B).

The DAPI staining results were supported and confirmed by the DNA quantification assay. As shown in Figure 2C, both cryopreservation and decellularization guaranteed a reduction in DNA content versus the NativeT group. Attributing to the NativeT group a DNA amount of 100% (522.30 ± 157.70 ng/mg), CryoT and DecellT samples showed a reduction of 60.4% (206.21 ± 71.26 ng/mg) (*p* < 0.0001) and 91% (55.84 ± 7.06 ng/mg) (*p* < 0.0001), respectively. Despite both cryopreservation and decellularization allowing a significant DNA amount decrease, decellularization was more effective. In fact, a significant difference in total DNA was detected comparing the CryoT and the DecellT groups (**: *p* < 0.01).

### 3.2. Immunolocalization of Alpha-Gal Epitopes

**Figure 3:**
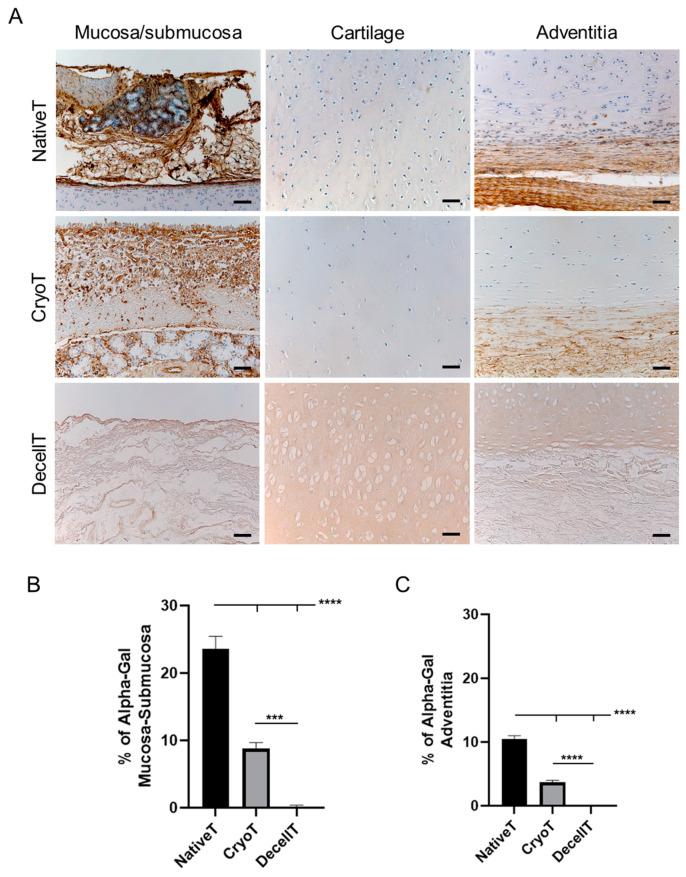
(**A**) Immunolocalization of Alpha-Gal epitopes in cryopreserved (CryoT) and decellularized (DecellT) samples versus native trachea (NativeT) (control). The mucosa/submucosa, cartilage and adventitia were considered separately. Positive elements are stained in brown. Scale bars: 50 µm. (**B**,**C**) Quantification of Alpha-Gal positive elements within the mucosa–submucosa and adventitia. (***: *p* < 0.001; ****: *p* < 0.0001).

To assess if cryopreservation and decellularization can modulate the distribution pattern of xeno-antigens, immunohistochemistry was adopted to describe Alpha-Gal epitope presence. This evaluation is fundamental when considering pig-derived CryoT or DecellT for an implant in humans. As shown in Figure 3A, Alpha-Gal positive elements were broadly detectable in NativeT at the level of mucosa and submucosa, mainly in correspondence of the glands; some positive lining cells were also identified at the interface cartilage/submucosa and cartilage/adventitia. Conversely, Alpha-Gal was nearly not recognizable within cartilage. CryoT, despite reduction, still maintained a certain immunopositivity for the antigen, especially in correspondence with the mucosa and submucosa. No positive reaction was highlighted in the whole DecellT thickness.

Alpha-Gal immunoreactivity was quantified with a focus on mucosa/submucosa (Figure 3B) and adventitia (Figure 3C). CryoT showed an immunoreactivity percentage of 8.76 ± 0.93% and 3.68 ± 0.33% at the mucosa/submucosa and adventitia side, respectively; these values were higher than that quantified for the DecellT group (mucosa/submucosa: 0.20 ± 0.18% (*p* < 0.001); adventitia: 0.02 ± 0.031% (*p* < 0.0001)) but lower when compared to the NativeT (mucosa/submucosa: 23.56 ± 1.87% (*p* < 0.0001); adventitia: 10.46 ± 0.54% (*p* < 0.0001)). As expected, NativeT distinguished within the cohort for Alpha-Gal immunoreactivity highest values at both the analyzed aspects of the tracheal tissue (*p* < 0.0001).

### 3.3. Histological and Biochemical Analyses

The CryoT maintained the typical microscopic structure of the NativeT with partial modifications likely descending from freezing and thawing. In the respiratory epithelium, only a few sparse cilia were detectable; the mucosa and submucosa tissues appeared looser than in the NativeT but the mixed glands were still recognizable and structurally intact. Focusing on cartilage, the tissue maintained its compact matrix organization. Despite the chondrocytes still being present, there were also void lacunae. The territorial matrix was still dark stained, similarly to NativeT; whereas the adventitia, even though identifiable, appeared looser and less organized than in NativeT.

The decellularization protocol partly modified the histological appearance of the tracheal segments by removing the cellular elements from the whole tissue thickness. After 12 treatment cycles, the respiratory epithelium disappeared. The mucosal and submucosal layers with the glands were not recognizable; only disorganized connective tissue remnants were identifiable. Considering the cartilaginous compartment, no cells were detectable within the lacunae which appeared wider than in the NativeT and CyoT, probably as consequence of the mechanical stress descending from lyophilization. Despite being lighter, staining of the territorial matrix was preserved. Decellularization also altered the adventitia, appearing as less organized than in the NativeT and CryoT groups.

**Figure 4:**
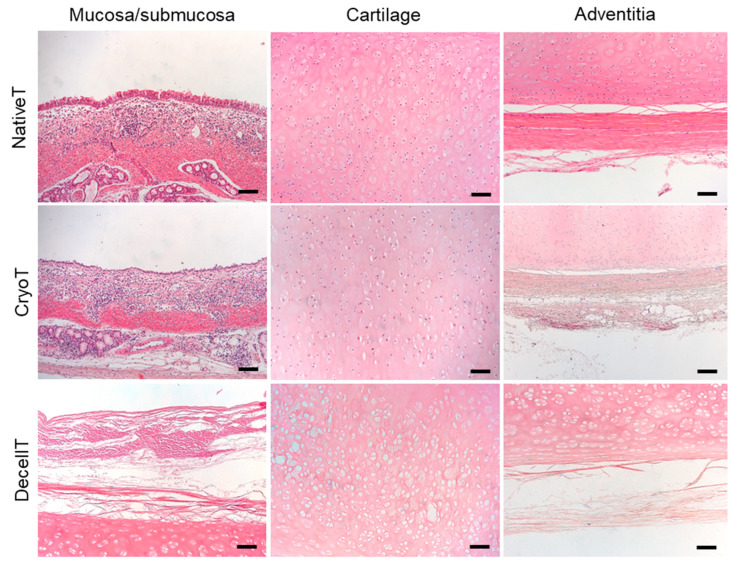
Histological appearance of pig tracheas obtained by cryopreservation (CryoT) and decellularization (DecellT) compared with native trachea (NativeT) showed by haematoxylin and eosin (H&E) staining. The whole tissue thickness was considered, highlighting the microscopic organization of the mucosa/submucosa, cartilage and adventitia. Scale bars = 100 μm.

Together with a description of tissue organization, H&E staining further supported qualitative/quantitative data from DAPI staining and DNA content analysis allowing for cell nuclei detection. As previously showed, a reduction in immunogenic elements (cells) was evident in CryoT; whereas, no cells were detected in DecellT (Figure 4).

**Figure 5:**
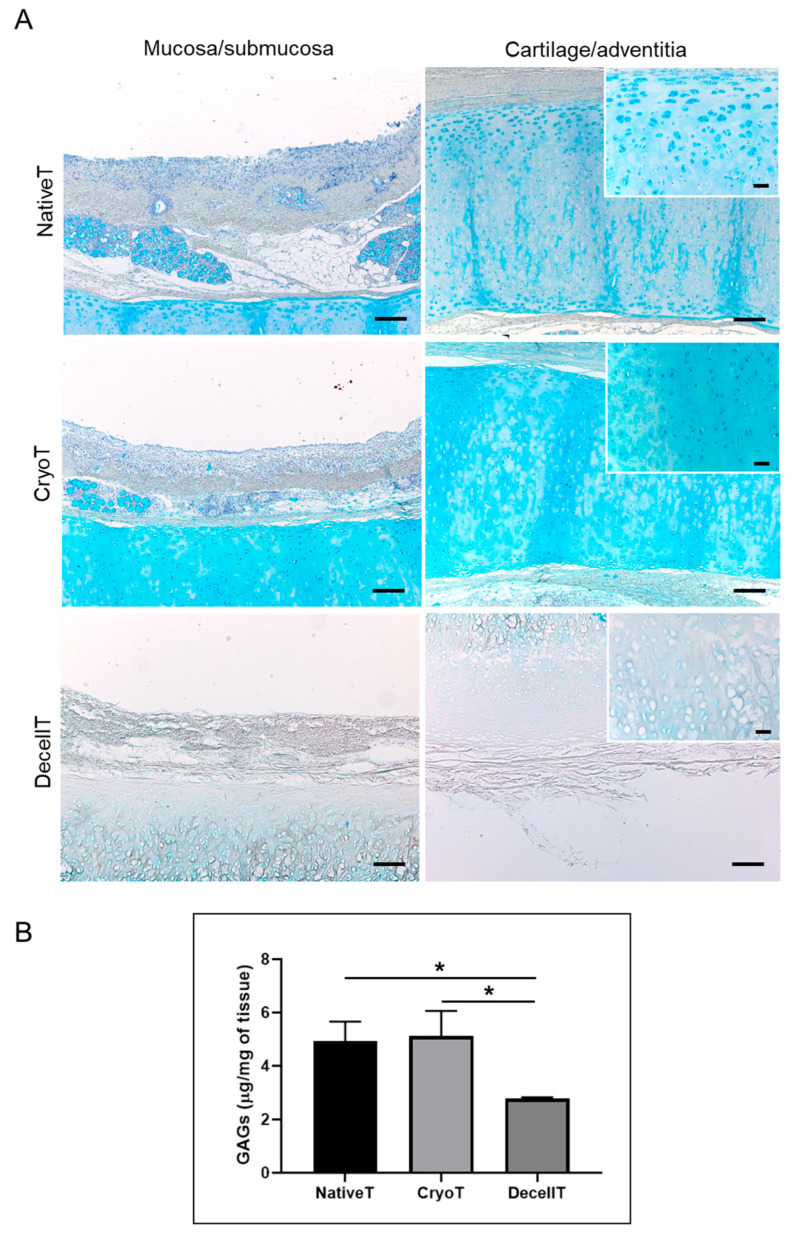
(**A**) Histological appearance of pig tracheas obtained by cryopreservation (CryoT) and decellularization (DecellT) compared with native trachea (NativeT) showed by Alcian Blue staining. The whole tissue thickness was considered. Scale bars: 100 µm (mucosa/submucosa); 50 µm (cartilage/adventitia); 200 µm (cartilage/adventitia, insert). (**B**) Sulphated glycosaminoglycans (GAGs) quantification, showing a reduction in GAGs content after decellularization (DecellT) compared with cryopreserved tracheas (CryoT) and native tracheas (NativeT) (*: *p* < 0.05).

Within each group, the glands secretory cells and the ECM were characterized by Alcian Blue staining showing cytoplasmic granules and GAGs presence, respectively. NativeT and CryoT samples displayed a similar blue staining. Conversely, a less intense blue colour was detected in the DecellT samples, suggesting glands’ modification and a certain GAGs depletion in cartilage ECM (Figure 5A). Typically, in the cartilaginous compartment, GAGs are mainly located in the territorial matrix: this was evident observing NativeT and CryoT samples. In accordance with preliminary H&E, DecellT territorial matrix was only faintly blue colored compared with the other experimental groups.

The biochemical assay corroborated the microscopic evidences. CryoT showed a mean GAGs content similar to that displayed by the NativeT (5.21 ± 0.90 μg/mg versus 4.93 ± 0.60 μg/mg). Conversely, decellularization was responsible for a significant (*p* < 0.05) loss of GAGs content (2.79 ± 0.04 μg/mg) versus the NativeT and the CryoT groups; despite that, the 56.4% of the initial GAGs amount was maintained (Figure 5B).

**Figure 6:**
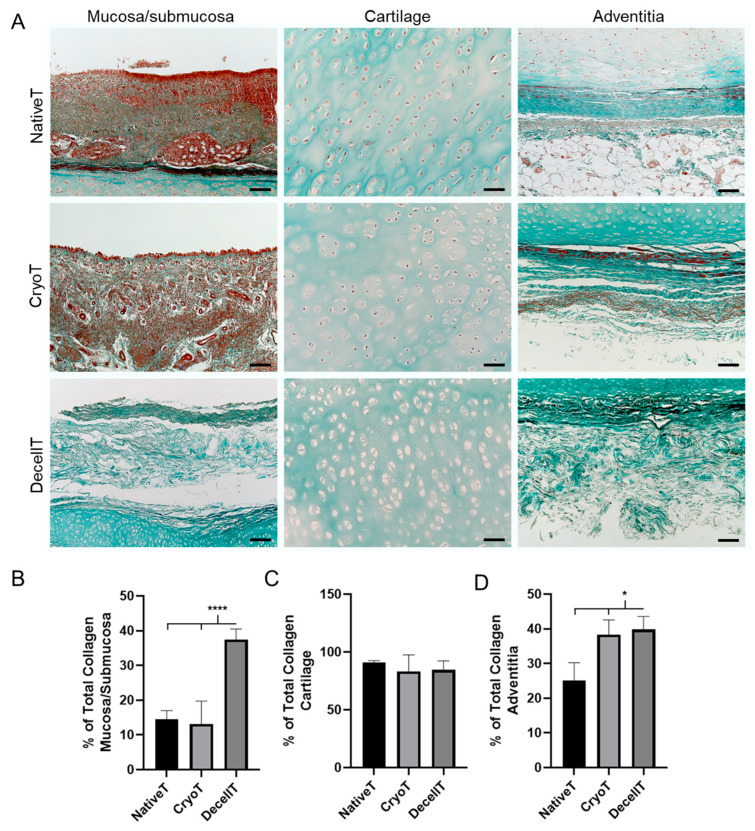
(**A**) Histological appearance of trachea cartilage tissue. Native trachea (NativeT), cryopreserved trachea (CryoT) and decellularized trachea (DecellT) were compared after Goldner Masson’s Trichrome staining for collagen fibers. was here focused. Scale bars = 100 μm (upper row); 200 μm (lower row). (**B**–**D**) Collagen content quantification within the mucosa/submucosa, cartilagineous compartment and adventitia. (*: *p* < 0.05; ****: *p* < 0.0001).

Collagen content was evaluated through different histological characterization studies. After Masson’s trichrome staining (Figure 6A), the mucosa/submucosa, the cartilage compartment and the adventitia were focused and quantification analysis was performed (Figure 6B–D). The green staining colour was uniformly represented, suggesting the presence of a broadly distributed collagen component in the hyaline cartilage. This evidence was also confirmed by green intensity quantification: the calculated mean values were 90.71 ± 1.79% for NativeT; 83.27 ± 14.30% for CryoT and 84.41 ± 7.95% for DecellT. No statistically significant differences were detected (Figure 6C). Differently, statistically higher values in total collagen were calculated for the mucosa/submucosa compartment and the adventitia in the DecellT group versus the other two groups (mucosa/submucosa: DecellT: 37.48 ± 2.99%; NativeT: 14.44 ± 2.59%; CryoT: 13.12 ± 6.61% (*p* < 0.0001). Adventitia: DecellT: 39.83 ± 3.73%; CryoT: 38.37 ± 4.18%; NativeT: 25.07 ± 5.23% (*p* < 0.05)) (Figure 6B,D).

**Figure 7:**
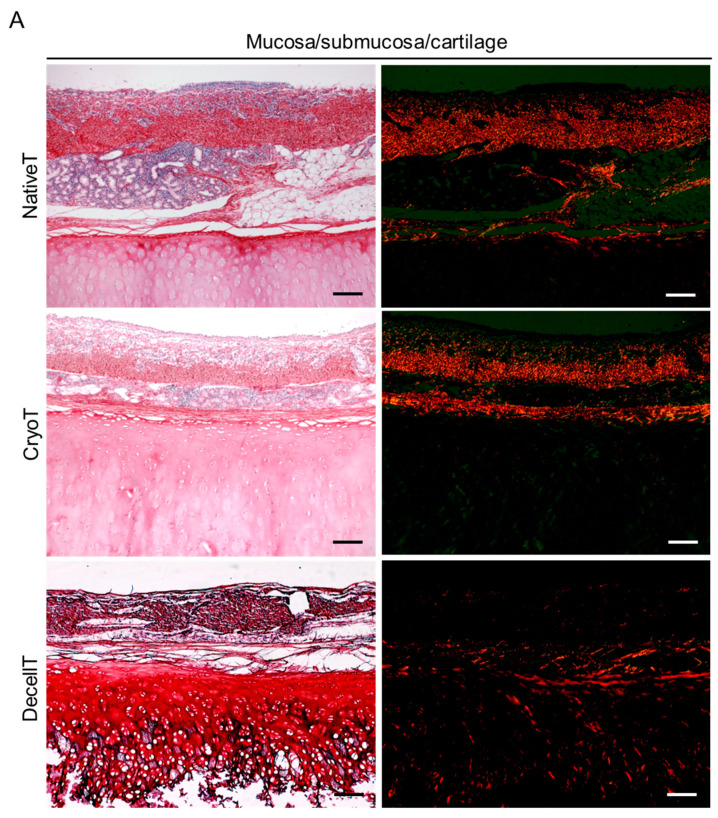

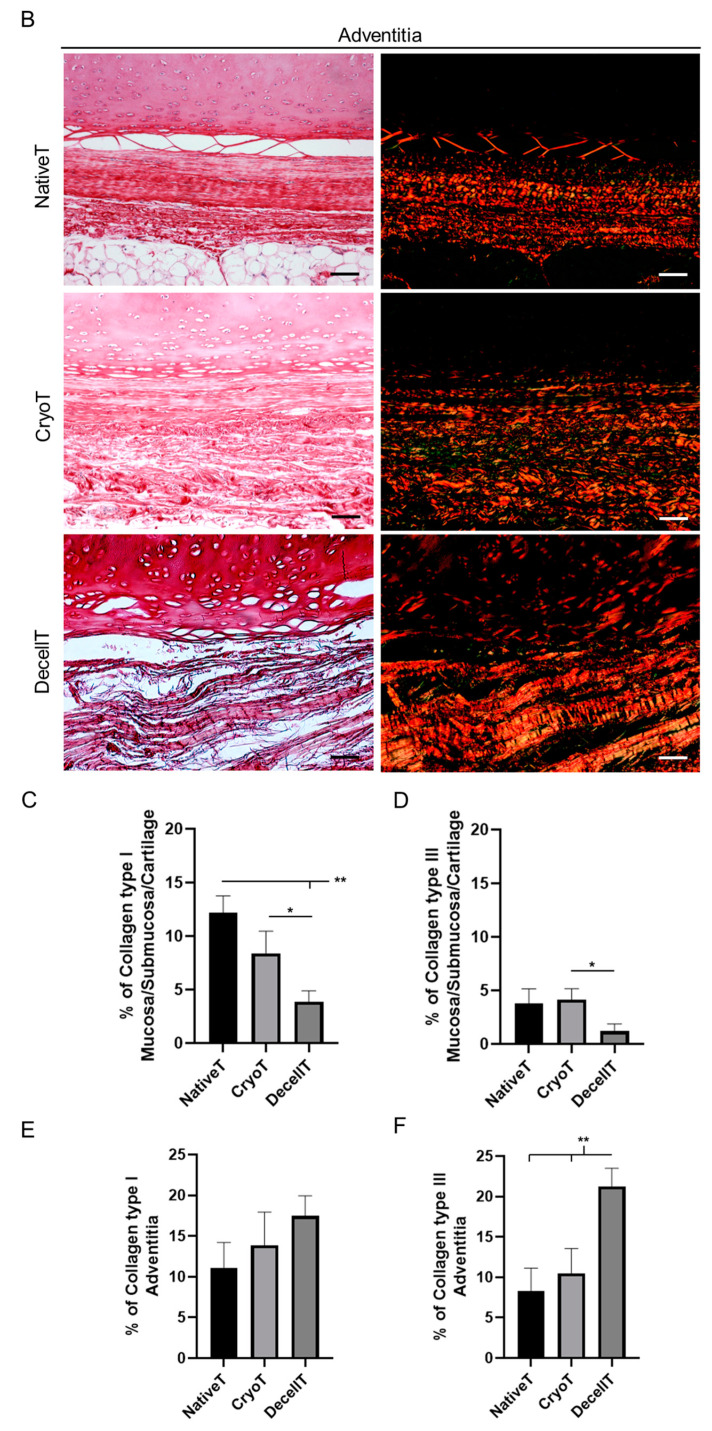
Histological appearance of pig tracheas obtained by cryopreservation (CryoT) and decellularization (DecellT) compared with native tracheas (NativeT) showed by Picrosirius Red staining and visualized under optical microscope (left column) and optical microscope in polarized light (right column). Taking advantage from bifringence, type I or type III collagen fibers were detected appearing as red-orange or green-yellow, respectively. The whole tissue thickness was analyzed: (**A**) mucosa/submucosa and cartilage; scale bars: 50 µm; (**B**) adventitia; scale bars = 100 μm. Quantification of collagen type I and type III within (**C**,**D**) the mucosa/submucosa/cartilage and (**E**,**F**) the adventitia (*: *p* < 0.05; **: *p* < 0.01).

Contextually, Picrosirius Red staining allowed to specifically detect collagen type I (orange-red) and type III (green) fibers under polarized light. Specifically, bifringence allowed to recognize a broad collagen type I presence (larger fibers) in both the mucosa/submucosa and perichondrium for the NativeT and CryoT groups; conversely, the DecellT samples displayed collagen type I fibers at the perichondrium but also within the cartilagineous compartment, at the level of the territorial matrix. Collagen type III (thinner fibers) was nearly not identifiable here (Figure 7A).

Similarly, also the adventitia distinguished for preferential localization of collagen type I fibers than collagen type III; however, this latter one was slightly more represented here than in the mucosa/submucosa (Figure 7B). Dark areas corresponded to portions revealing less density in collagen fibers.

Prevalence of collagen type I and III was determined at both the mucosa/submucosa/cartilage and adventitia side. Focusing on the mucosa/submucosa/cartilage compartment, CryoT samples showed a higher content in collagen type I than the DecellT group (8.41 ± 2.05% and 3.85 ± 1.04%, respectively; *p* < 0.05). A statistically significant difference (*p* < 0.0001) was also calculated between NativeT (12.21 ± 1.54%) and DecellT. As regards collagen type III, NativeT and CryoT showed a comparable amount in this protein (3.81 ± 1.34% and 4.15 ± 1.01%, respectively); a significant difference was only identified between CryoT and DecellT (1.20 ± 0.68%) (Figure 7C,D). Regarding the adventitia, an opposite collagens preponderance was recognized here versus the mucosa/submucosa/cartilage side. In fact, the DecellT group showed the highest calculated values in collagen type I and type III than the other groups. While the difference was not statistically significant for collagen type I (NativeT: 11.08 ± 3.14%; CryoT 13.89 ± 4.06%; DecellT 17.46 ± 2.48%), a *p* < 0.01 aroused comparing DecellT (21.22 ± 2.26%) with CryoT (10.48 ± 3.08%) and NativeT (8.32 ± 2.82%), respectively (Figure 7E,F).

**Figure 8:**
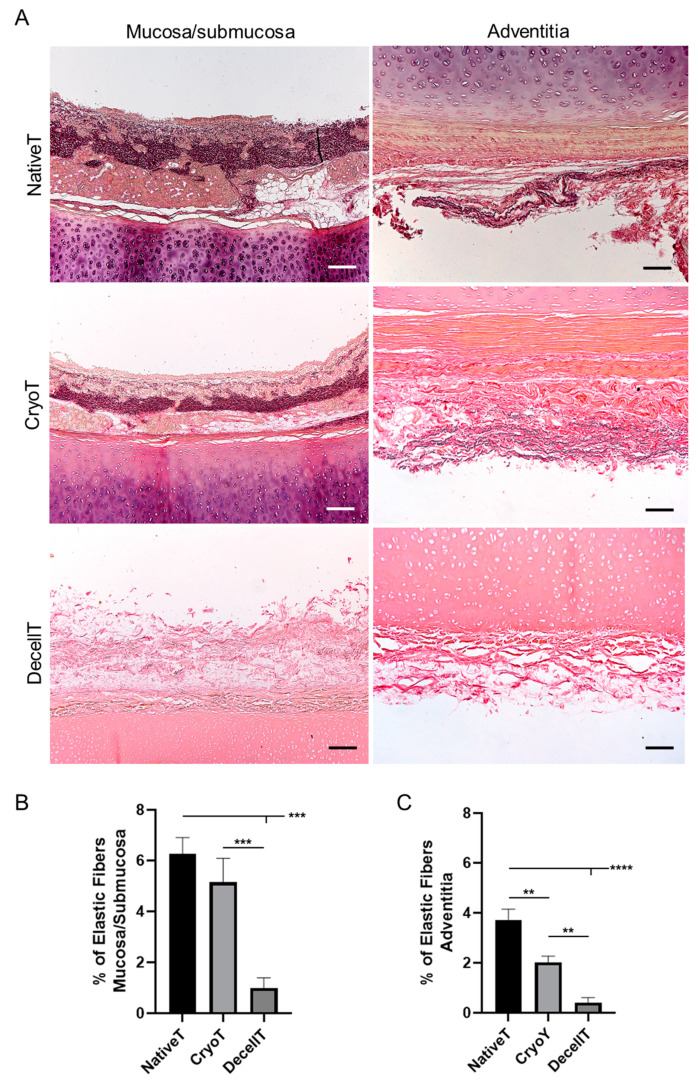
(**A**) Histological appearance of pig tracheas obtained by cryopreservation (CryoT) and decellularization (DecellT) compared with native tracheas (NativeT) showed by Weigert Van Gieson staining highlighting presence and distribution of elastic fibers (purplish elements). The mucosa/submucosa and adventitia were focused. Scale bars: 50 µm (mucosa/submucosa); 100 μm (adventitia). (**B**) Elastic fibers content within the mucosa/submucosa and (**C**) adventitia. (**: *p* < 0.01; ***: *p* < 0.001; ****: *p* < 0.0001).

Considering the elastic fibers, their presence and distribution was compared among the experimental groups to verify eventual modifications ascribable to cryopreservation or decellularization. Typically, as showed in NativeT, the elastic fibers form the lamina elastica (separating the mucosal from the submucosal layers) where they are densely packed as longitudinally running elements. Besides the lamina elastica, they can also be detected in subepithelial position, as a sheath; at the surface of glands, organized in a net; in close contact to the inner surface of the cartilaginous ring, condensed at the deep border and outside the cartilage, within the adventitia. While the CryoT samples showed to maintain the elastic fibers pattern, a different appearance was displayed by DecellT tissues. Decellularization affected the elastic fibers content whose remnants were partly identifiable as slightly stained elements at the lamina elastica and at the deep and outer layers of the cartilaginous ring (Figure 8A).

The elastic fibers content within the mucosa/submucosa (Figure 8B) and adventitia (Figure 8C) was determined for a comparison in terms of prevalence among the three groups. CryoT showed at both sides (mucosa/submucosa: 5.15 ± 0.94%; adventitia: 2.02 ± 0.25%) lower values than NativeT (mucosa/submucosa: 6.67 ± 0.64%; adventitia: 3.72 ± 0.43% (*p* < 0.01)) but a higher content in elastic fibers than the DecellT group (mucosa/submucosa: 0.99 ± 0.41% (*p* < 0.001); adventitia: 0.42 ± 0.19% (*p* < 0.01)). As expected, NativeT distinguished over DecellT at both sides (mucosa/submucosa: *p* < 0.001; adventitia: *p* < 0.0001).

### 3.4. Ultrastructure and Collagen Fibers Organization

**Figure 9:**
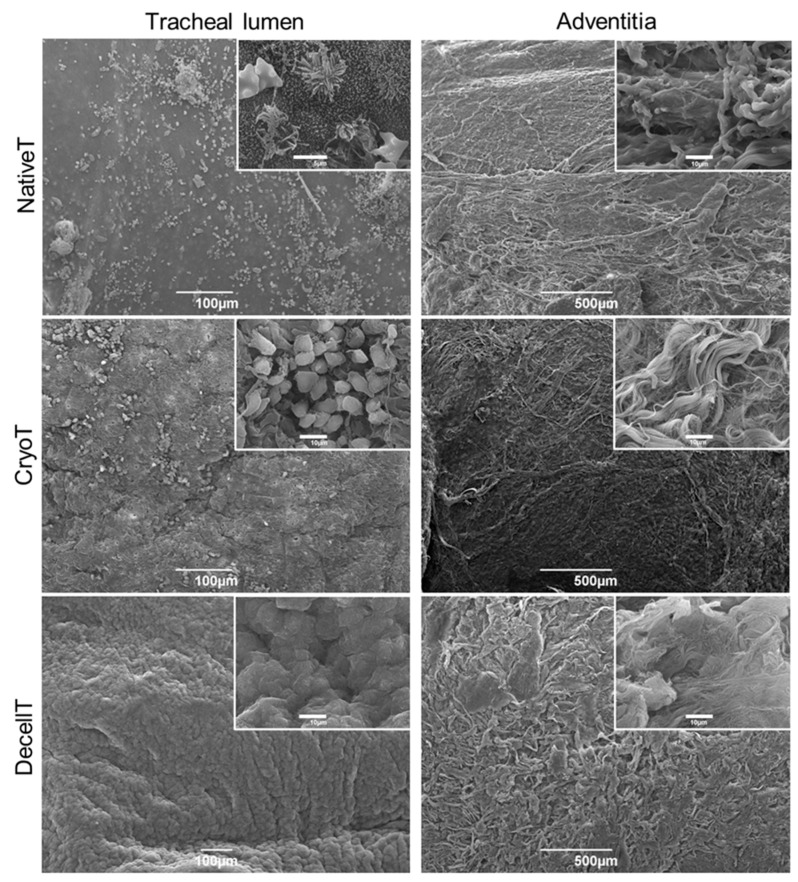
Representative photomicrographs showing ultrastructural appearance of both tracheal lumen and adventitia for native trachea (NativeT), cryopreserved trachea (CryoT) and decellularized trachea (DecellT). Scale bars: 100 µm (tracheal lumen); 500 µm (adventitia); 5 µm (NativeT tracheal lumen, insert); 10 µm (all other inserts).

The ultrastructure of both the respiratory epithelium and adventitia was investigated by SEM (Figure 9). The NativeT luminal surface showed the typical appearance of the intact respiratory epithelium characterized by cilia and microvilli. Conversely, a different ultrastructure was associated with CryoT and DecellT samples; in fact, despite in a different manner, cryopreservation and decellularization showed to impact the respiratory epithelium integrity. A certain de-epthelization was identified in the CryoT group, respiratory cells lost cilia or cilia completeness and only few remnants were recognizable; in some portions, it was possible to identify basal lamina fibrillae. Regarding the DecellT specimens, the protocol adopted led to respiratory epithelium cells removal, suggesting they were peeled-off by physical+enzymatic+chemical approach. Although DecellT showed a complete removal of the epithelium, basal membrane with a smooth and convoluted appearance was still detectable. No significant differences were recognized between the NativeT and the CryoT in the adventitia; both displayed finely organized collagen fibers, clearly identifiable even after cryopreservation. Differently, the collagen fibers were still identifiable in the DecellT samples (at higher magnification) but they appeared as partially fused, likely as consequence of the treatment they were exposed to.

**Figure 10:**
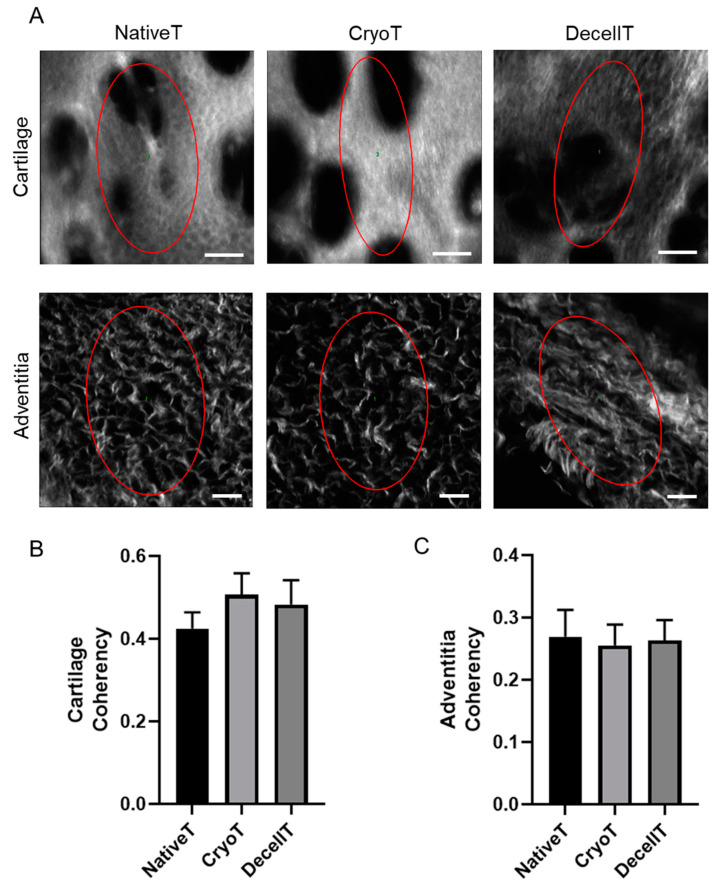
Evaluation of collagen fibers distribution into the cartilaginous compartment and adventitia side of cryopreserved and decellularized tracheas (CryoT and DecellT, respectively) versus native trachea (NativeT) used as reference. (**A**) Second Harmonic Generation (SHG) signal is showed in gray; in accordance with the Fast Fourier Transforms (FFTs), the ellipsoidal fibre orientation, suggesting anisotropy, is described by the red ellipse profile inside each photomicrograph. (**B**,**C**) Average values of collagen Coherency, which estimates the local orientation of the fibres in the cartilage (0.42 ± 0.04; 0.51 ± 0.05; 0.48 ± 0.06 for NativeT, CryoT and DecellT respectively) and adventitia (0.27 ± 0.04; 0.25 ± 0.03; 0.26 ± 0.03 for NativeT, CryoT and DecellT respectively); coherency was calculated using the ImageJ plugin OrientationJ. No significant difference was identified among groups. Scale bar: 20 µm.

In parallel, tissue fibers spatial organization was described by SHG microscopy. Like NativeT, the CryoT and DecellT groups showed collagen fibers mainly oriented in one direction (ellipsoidal shape for FFT) (see the three red elliptic profiles in Figure 10A) in both the compartments (cartilage and adventitia), suggesting an anisotropic behaviour. No significant difference was detected among groups regarding both cartilage and adventitia (Figure 10B,C).

### 3.5. Compressive Mechanical Behavior

In order to compare the compressive mechanical behaviour of different trachea samples, their size and shape were first analyzed. The obtained measurements are reported in Table 1. These data show that all the samples had homogenous sizes in DecellT, CryoT and NativeT segments, with only few differences in the medio-lateral diameter of DecellT samples, which was slightly higher than the other groups.

**Figure 11:**
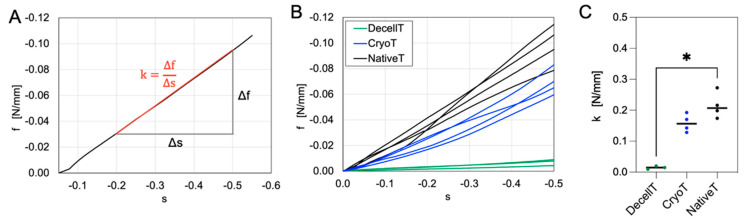
Results of compression tests: (**A**) Typical compression curve in terms of force per unit of length of the sample f vs. tracheal compressive deformation s in the proximal-distal direction. The compressive modulus k is the slope of the secant line between 20% and 50% of compressive deformation. (**B**) Compression curves of all the testes samples of DecellT, CryoT and NativeT. (**C**) Comparison of stiffness among all the testes samples of DecellT, CryoT and NativeT (*: *p* < 0.05).

The results of the compression tests, carried out on DecellT, CryoT and NativeT, are summarized in Figure 11. The typical trend (Figure 11A), reported in terms of force per unit of length of a sample *f* vs. tracheal compressive deformation *s* in the proximal-distal direction, showed an initial region, below 10% of deformation, with lower stiffness, followed by a quasi-linear region with increased stiffness up to the highest reached compression. This change of the slope in the compression curve was due to a variation of contact area during the test. Indeed, since the sample diameter was not constant all over its length, just one of the cartilaginous rings was in contact with the upper plate at the beginning of the test. While the upper plate was approaching the samples, the other cartilaginous rings came in contact with it. This typically occurred in the range between 5% and 20% of deformation. The contact has then become homogenous on the overall sample length and the compressive behaviour was almost linear. The compressive stiffness of the trachea samples was therefore evaluated in this region, considering the secant modulus between 20% and 50% of compressive deformation. The experimental data in Figure 11B show that the compressive behaviour of NativeT and CryoT was very similar, while the DecellT could be occluded up to 50% of compressive deformation with a much lower force per unit of length. The comparison among compressive stiffness values (Figure 11C) highlighted a significant difference between DecellT and Native T samples.

### 3.6. Cytocompatibility Assessment In Vitro

**Figure 12:**
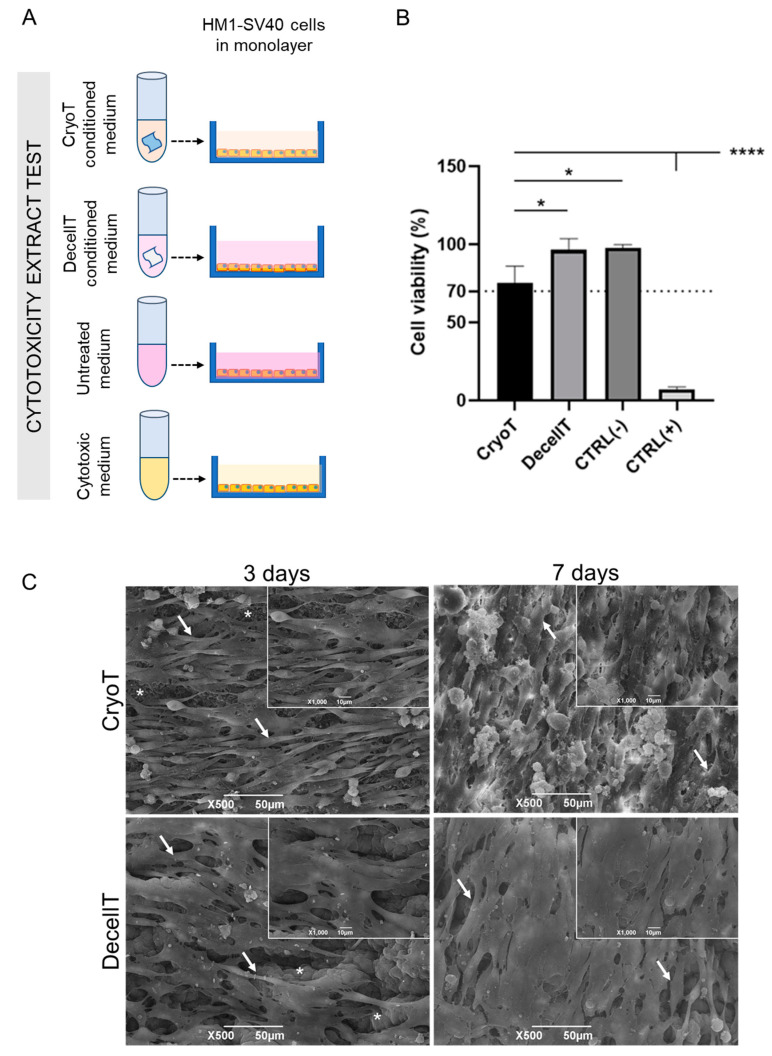
Cytocompatibility evaluation through the indirect and the direct methods. (**A**) Schematic figure showing the methodological approach to perform the cytotoxicity extract test. (**B**) test results on HM1-SV40 cell line. The cells were incubated for 24 h with culture media previously conditioned with cryopreserved and decellularized trachea (CryoT and DecellT, respectively). For each group, cell viability percentage was determined by comparison with that of untreated cell cultures, set at 100% of viability (*: *p* < 0.05; ****: *p* < 0.0001). (**C**) SEM analysis of cell-scaffold interactions in terms of cell attachment, morphology and proliferation on tracheal matrices. Scale bar: 50 µm; 10 µm (top right insert).

The in vitro cytocompatibility study, developed as schematically reported in Figure 12A, allowed to predict biocompatibility in vivo, excluding possible undesired reactions amenable to chemicals retains. After 24 h of culture with proliferative medium previously conditioned with CryoT and DecellT, the HM1-SV40 cells showed to preserve typical morphology, viability and proliferative behaviour, reaching about 50–60% confluence on the growth surface. The MTT analysis also supported optical microscopy data.

Compared to untreated cultures (CTRL-) (viability set at 100%), cells growth in CryoT and DecellT conditioned medium was 77.3% and 98.9%, respectively (*p* < 0.05). Both values remained above the reference threshold value (70% cell viability) which is required to consider a sample as non-cytotoxic. As expected, significant differences (*p* < 0.0001) were detected between the cytotoxic control and other groups. Interestingly, higher viability was observed for cells treated with the DecellT conditioned medium than the CryoT ones (*p* < 0.05) (Figure 12B).

Scaffold cytocompatibility was further demonstrated by HM1-SV40 cell seeding on cryopreserved and decellularized tracheas. SEM micrographs reported in Figure 12C show that both samples were colonized by seeded cells since day 3 from seeding, with HM1-SV40 populations exhibiting the typical fibroblastoid morphology on scaffolds. At day 7 cells seemed to be more numerous on both samples, suggesting adequate proliferation over time. On CryoT supports, cells appeared to grow more rapidly, reaching over confluence, and starting to detach from the seeding surface.

### 3.7. Biocompatibility Assessment In Vivo and Explants Characterization

**Figure 13:**
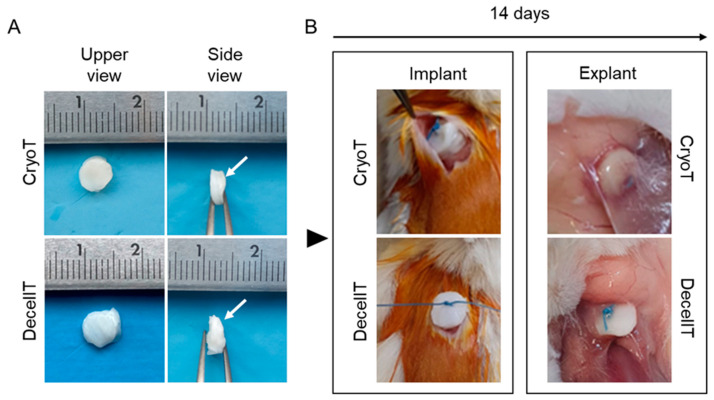
Gross appearance of cryopreserved (CryoT) and decellularized (DecellT) trachea samples before implant (the white arrows show the mucosa side) (**A**), after in vivo positioning in Balb/C mice (subcutaneous dorsal pouch) and at retrieval (14 days from surgery) (**B**). The mucosa side was placed in contact with the *latissimus dorsi*.

At surgery the two groups’ samples displayed a similar gross appearance; when anchoring the specimens, no rupture or layers decoupling occurred. The mucosal layer was easily identifiable in both CryoT and DecellT samples, thus allowing for adequate positioning in vivo (tracheal mucosa in contact with the *latissimus dorsi*). No decoupling occurred between the two aspects during sampling and/or surgery manipulation (Figure 13A).

At 14 days from implant, all the specimens were clearly identifiable at retrieval, without giving rise to dislocations or severe inflammatory reactions; only a thin fibro-connective capsule was detectable surrounding the CryoT scaffolds (Figure 13B).

**Figure 14:**
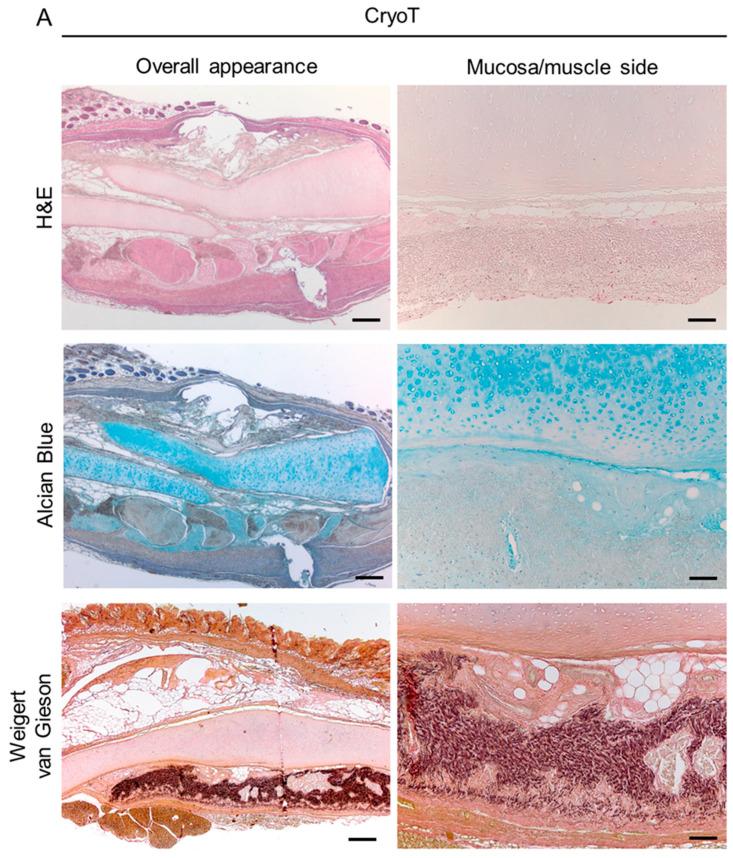

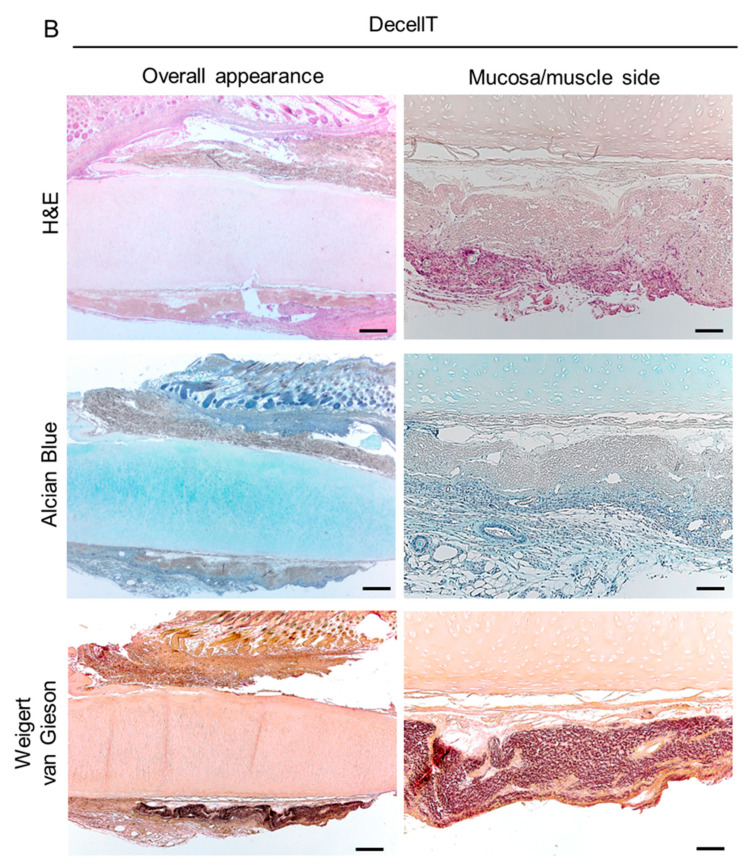
Histological characterization of the explants by haematoxylin end eosin (H&E), Alcian Blue and Weigert van Gieson stainings. Both the overall appearance of the explants with the surrounding tissues and the mucosa/submucosa side were highlighted for the cryopreserved (**A**) and decellularized (**B**) tracheas (CryoT and DecellT, respectively). Scale bars: 400 µm (overall appearance); 100 µm (mucosa/muscle side).

The histological analyses supported the macroscopic evidence at dissection (Figure 14A,B). No alteration occurred within the CryoT and the DecellT samples, at the level of the cartilagineous compartment, which remained intact and well recognizable in all the sections (H&E and Alcian Blue stainings). Considering the external borders of the implants, a slight host reaction was evident in both groups, compatible with the type of surgery performed. In particular, the connective sheath, also characterized by elastic fibers presence (Weigert Van Gieson), was similar in CryoT and DecellT at the subcutis/cartilage interface but thicker in CryoT than in DecellT at the mucosa/*latissimus dorsi* muscle contact area.

**Figure 15:**
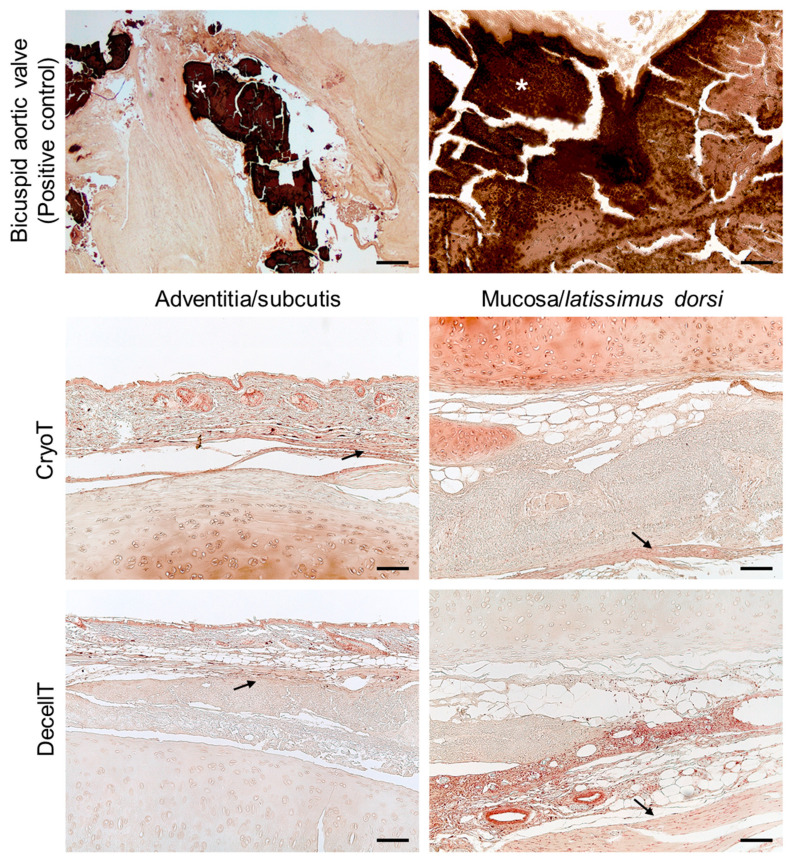
Histological characterization of the CryoT and DecellT explants by von Kossa staining. Both the mucosa/submucosa side and adventitia, in contact with the subcutis and the latissimus dorsi, respectively, were focused (the back arrow shows the interface) (scale bars: 100 µm) A positive control (calcification of bicuspid aortic valve, white asterisk (*)) was included in the first row (scale bars from the left to the right: 100 µm; 50 µm).

No detection of positive reaction after von Kossa staining (black deposits) highlighted that neither cryopreservation nor de-cellularization affected calcification of the samples after in vivo implant (Figure 15). Both the subcutis/cartilage interface and the mucosa/*latissimus dorsi* muscle contact area were analyzed.

**Figure 16:**
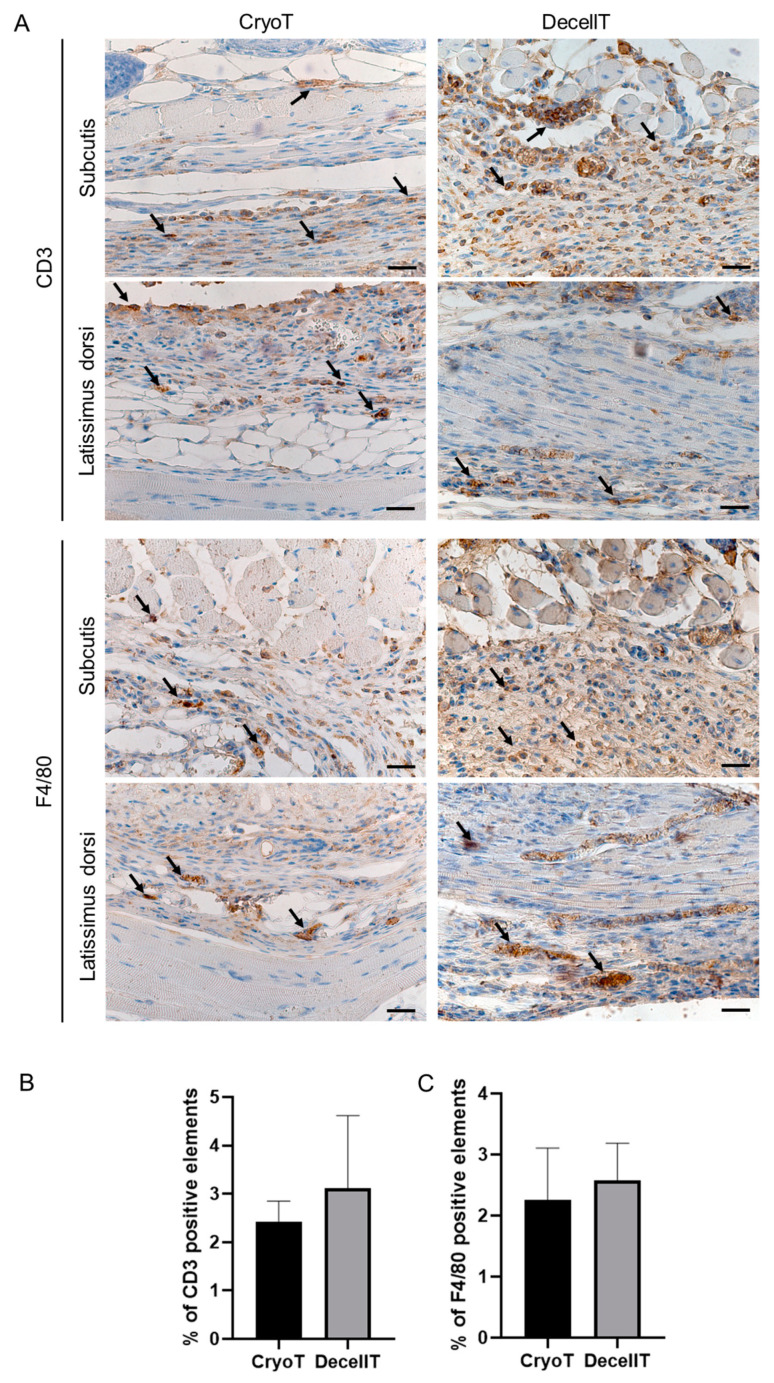
(**A**) Immunohistochemical characterization of cryopreserved (CryoT) and decellularized (DecellT) samples after heterotopic positioning in Balb/C mice subcutaneous pouch and retrieval at day 14 from surgery. The immunolocalization of CD3+ and F4/80+ cells (dotted brown-stained elements, black arrows) at the boundaries between the tracheal specimens and the host tissues showed the presence of a mild lympho-monocytic infiltration triggered by the scaffolds. Scale bars: 25 µm. Quantification of CD3 and F4/80 positive elements (**B**,**C**) within the CryoT and DecellT samples, after retrieval. No significant difference was detected.

Immunohistochemistry further confirmed histological characterization study data, proving the presence of T lymphocytes (CD3 positive elements) and monocytes/macrophages (F4/80 positive elements) in correspondence of the connective tissue surrounding the implants, mainly at the muscle side (Figure 16A). Immune reaction severity was mild; in addition, the infiltrate was more evident in correspondence of the stiches, suggesting a certain physiological response to suture. Quantification of CD3 and F4/80 positive elements showed no significant difference between the CryoT (CD3: 2.43 ± 0.42%; F4/80: 2.26 ± 0.85%) and the DecellT (CD3: 3.11 ± 1.50%; F4/80: 2.58 ± 0.61%) groups (Figure 16B).

**Figure 17:**
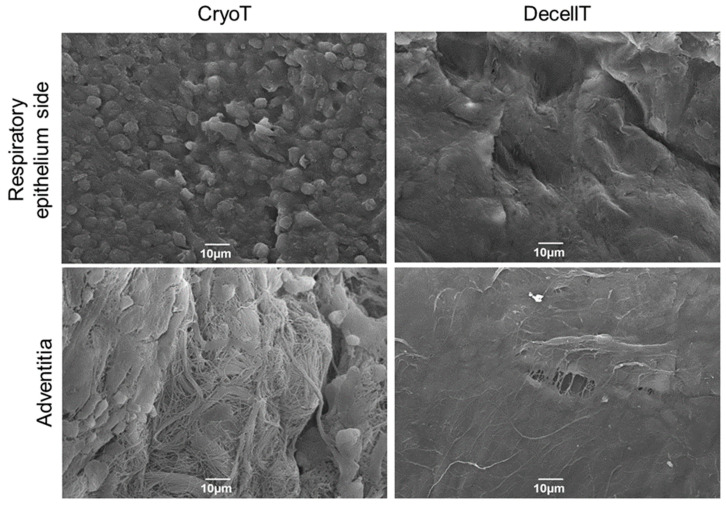
Ultrastructural characterization of retrieved cryopreserved (CryoT) and decellularized (DecellT) samples after heterotopic implant in Balb/C mice (end-point: 14 days). The respiratory epithelium side and the adventitia were both considered. Scale bars: 10 µm.

To broadly characterize the explants, an ultrastructural analysis was also performed. The CryoT samples showed the presence of numerous cellular elements at the respiratory epithelium side. These were fused in a monolayer or roundish. Conversely, few host cells were evident at the adventitia side, adhering to collagen fibers. In a different manner than CryoT, the DecellT samples were broadly colonized by the host cells in the two sides; cells monolayers were clearly distinguishable, and no scaffold ultrastructure was evident (Figure 17).

## 4. Discussion

In case TRA is not feasible, identification of effective transplantation options, free from need of immunosuppressant therapies to preserve grafted tissue structural/functional integrity, are an ambitious goal to pursue. Together with eliminating immunosuppressant therapy side effects and the related costs, they would guarantee for significant improvements of patients’ life quality, especially (but not limited) to the oncologic category [11,56,57,58].

To date, several studies regarding fabrication of tubular substitutes to restore trachea continuity have been encouraged by the apparent simplicity of the “windpipe” [11] but a resolutive reconstructive option is still lacking, as suggested by the intense research still ongoing in this field [10]. Patent grafts development and integration after positioning, with also respiratory epithelium recovery, is fundamental to guarantee for a native-like tissue regeneration (instead of reparation) whose functions by far exceed the simple conduction and conditioning of air [59]. It descends that trachea allograft (or even xenograft) are possibly the most compliant option to resort to, as suggested by derivative’ adequate macroscopic and microscopic anatomy, ECM proteins type/content and ultrastructural organization [60,61,62].

Given the shortage of human donor material for allotransplantation, animal-derived tissues enlarge the options for treatment, being used directly as a transplant (xenotransplant, xenograft) after processing like cryopreservation, or being prepared as a decellularized ECM. These products intended for biomedical applications are regulated either as medicinal products or as medical devices. Specifically, in Europe, the Directive 2001/83/EC13 and Regulation (EC) No. 726/200414 regulate the use of xenogenic materials as medicinal products. In addition, the Regulation (EC) No. 1394/2007/EC15 has been approved as a *lex specialis* regarding advanced therapy medicinal products (ATMP). On the other hand, animal-derived materials for transplantation are regulated also as medical devices according to the Regulation No. 2017/745/EU. For xenogeneic products, further guidance information for general safety and risk assessment are provided by the *CHMP Guideline on xenogeneic cell-based medicinal products*, whereas the WHO Changsha Communique and follow-up documents have been approved to guarantee regulation and guidance on animals, donor source, processing, risk management, and safety evaluation (reviewed by Godehardt and Tönjes, 2020 [63]).

### 4.1. Trachea Substitutes Development

In this work, pig-derived CryoT and DecellT substitutes were developed and compared in a broad study aiming to provide a useful structural description of these two alternatives for trachea reconstruction. Several cryopreservation and decellularization protocols were attempted over the years, since the first efforts [64,65,66]; however, due to the contradictory results reported, their translation to medicine is still limited [8,67], except for compassionate use [28,35,36].

Cryopreservation approaches depend on temperature freezing rate, cryoprotective agents, and duration of cryopreservation [7]. The first preclinical study (pig) reporting about cryopreserved tracheal graft implantation in orthotopic position was described by Lenot et al. [68]. Despite surgery failure due to inadequate blood supply, the histologic structures and the mechanical properties were well preserved, stimulating intense research on this substitute type [15,24,26,28,38,60,61,67,69,70,71,72,73,74,75,76,77,78,79,80,81,82,83,84,85,86,87,88,89,90,91,92,93,94,95,96,97]. Hence, focusing on freezing temperature and liquid nitrogen storage, cryopreserved tracheal graft protocols can be distinguished into two groups: (a) freezing at −60° to −140 °C and storage in liquid nitrogen until use; (b) freezing at −80°/−85 °C, without recurring to liquid nitrogen storage [24]. The first strategy was here followed (freezing at −140 °C; storage in vapor phase liquid nitrogen), since long time adopted by the FBTV for successful cryopreservation of different other tissues used in clinical practice. These include, for instance, fascia lata allograft for surgical facial reanimation [39]; amniotic membrane for the treatment of gingival recessions [43] and cryptoglandular anal fistulas [98]; aortic homograft for aortic valve or aortic root replacements [22]. Interestingly, also decellularized human aortic valves underwent to this cryopreservation within an in vitro study [42].

Regarding tracheal decellularization, both physical + chemical methods [31,33,99,100,101,102,103] and physical + enzymatic + chemical methods [6,104,105,106,107,108,109,110,111,112,113,114] were approached and verified by several Authors considering orthotopic positioning. However, to date, no gold-standard strategy exists among decellularization protocols; the method can vary or be tuned along with tissue/sample characteristics mainly affected by the species of origin. Several factors, such as native tissue cells density, matrix thickness, lipid content, and species of origin (small or large animals) may influence decellularization efficiency [115]. To this purpose, evaluations on porcine trachea were performed, as it shows biomechanical properties similar to those of humans [116], providing for valuable data in perspective of implant on large animals as relevant translational model [117].

According to pre-clinical studies, miscellaneous protocols, combining chemical and enzymatic treatments to physical strategies, seem to be the preferred choice for effective trachea decellularization [6,104,105,106,107,108,109,110,111,112,113,114], including pig trachea [104,108,114]. Hence, this procedure was here followed adopting DNase, trypsin and Tergitol^TM^ detergent solution. DNAse helped in nucleic acid sequences cleavage and, therefore, in nucleotides removal. Its use was documented by several Authors that recurred to physical+enzymatic+chemical methods for trachea decellularization [6,104,105,109,110,111,112], eventually mixed with the Rnase [106,107,113]. The serine protease trypsin supported the complete elimination of cell nuclei from the dense tracheal ECM. However, the exposure was limited to 1 h (concentration: 0.05% *w*/*v*) being disruptive to elastin and collagen (despite showing better preservation of GAGs than sodium dodecyl sulphate (SDS)) in a time-dependent manner [34,118]. Focusing on detergents, Tergitol^TM^ was chosen. According to our knowledge, this is the first time that Tergitol^TM^ is adopted within a protocol for tracheal tissue decellularization. Others reported the use of sodium deoxycholate (SDC) [102,103,107,108,109,110,112], eventually combined with Triton X-100 [106,107,113]. SDC is an ionic surfactant leading to complete cell and nucleic membranes solubilization, with possible proteins’ denaturation. Triton X-100 is a non-ionic surfactant; recently, it was included by the European Chemicals Agency (ECHA) in the list of substances of very high concern of the Registration, Evaluation, Authorisation and Restriction of Chemicals (REACH) Regulation, due to its potential toxicity to the endocrine human system. Hence, the need for its replacement is urgent, driving towards the identification of a valid substitute.

Chemicals may impair the ECM characteristics; hence, a combination with physical techniques can ameliorate the interaction solution/tissue, while reducing the exposure time. Moreover, tracheal cartilage density, despite essential for a functional graft, is likely an obstacle to detergents/enzymes penetration [119]. In the past, cartilage tissue ultrastructure was sacrificed to finally develop an acellular suspension to combine with synthetic polymers in a functional composite scaffold [120,121]. Considering the importance of tissue structure preservation for trachea, lyophilization was here introduced before each cycle (typically, prior to soak the tissue in Dnase solution) to boost decellularization solutions’ penetration. As previously discussed for larynx by Hung et al. [122], improving tissue tendency to absorb fluids (as during rehydration after the freeze-drying) may enhance the method effectiveness. According to our knowledge, trachea lyophilization was firstly suggested in 1951, as a long-term preserving method prior to proceed with segment implant [123]. Later, it was introduced as an important decellularization step before trachea sonication in sodium dodecyl sulfate (SDS) solution [100] or within other complex decellularization protocols [124]. Osmotic shock through several washes in dH_2_O was also included; the aim was to trigger cell membranes lysis but also support enzymatic/chemical solutions removal avoiding retains within the ECM net.

### 4.2. Cryopreservation and Decellularization Decrease Immunogenicity

Cryopreservation of the tissues was protracted for 13 months, prior to proceed with the subsequent characterization analyses; decellularization took 12 weeks (12 cycles) to be completed, as corroborated by DAPI staining (later also confirmed by histology) and DNA quantification assay, revealing a—91% in DNA total content. This is a crucial fact because residual DNA fragments in decellularized ECM may lead to cytocompatibility issues in vitro and adverse immunological response upon implantation [125,126]. Likewise, also cryopreservation was associated with an important reduction in genetic material (−60.4%). These are intriguing data, considering the limited tissue manipulation required by the method. Cells were still recognizable in each of the tissue three-layers; however, cryopreservation showed its potential in the tissues’ antigenicity modification, suggesting that freezing periods modulation might further decrease the immunogenic potential of the tissue [8,25,74,88,97]. Intermittent immunosuppression was reported in preclinical studies [86], even though tracheas implant without recurring to immunosuppression, was also successfully assessed [72,74,80,89,96]. Freezing and thawing may induce a depletion and loss of class II antigen expression amenable to respiratory epithelium [24,76,79,91,96,127]. Together with these promising data, contrasting evidence also arose suggesting that no effect was exerted by prolonged periods of cryopreservation on the chondrocytes’ viability and thus tracheal allogenicity [83,85,92]. It is very important to confirm the immunomodulatory effect of cryopreservation on tracheal allografts to allow potential clinical application of tracheal transplantation in the future [8]. Intense research efforts focusing on freezing time, cooling speed, cryoprotectant type/mixture may guarantee for the development of effective, cost-saving and safe allografts [23], not eliciting adverse immune reaction but also growing without calcifications and remaining patent [89,90,97]. Within this scenario, xenografts may represent a valuable resource to bridge the gap between the supply and demand of organs/tissues; however, immunological barriers must be considered as xenotransplants are prone to rejection [128]. In particular, in case of wild-type pig organ transplantation into a human, a hyperacute rejection with graft destruction may occur and the major xenoantigen responsible of that is Alpha-Gal epitope [128,129]. In consideration of this, the effects mediated by cryopreservation and decellularization over Alpha-Gal epitope expression were analyzed. Compared to NativeT, CryoT still showed positive elements at the mucosa/submucosa layer and few remnants at the adventitia side, lining the cartilage. Conversely, the DecellT was Alpha-Gal epitope-free; morphometric analysis corroborated this evidence also highlighting a Alpha-Gal reduction after cryopreservation of about 37,18% and 35,18% in mucosa/submucosa and adventitia, respectively. The results provided by this study, despite only qualitative, are consistent with the literature. Cryopreservation may reduce the risk of immune rejection but cannot completely eradicate all the immune rejection inducing Alpha-Gal antigens [130]. Decellularization can be effective in removal of donor cells but also xenoantigens, including Alpha-Gal epitopes [131,132].

Regarding the immunogenicity of cryopreserved grafts after transplantation, clinical examination of patient immune response during the early postoperative course and long-term follow-up have scarcely been reported so far. Some trials investigated the immunogenicity of cryopreserved valved and nonvalved allografts used in the surgical repair of congenital heart defects, describing relevant HLA antibody response induced by transplanted materials [133,134,135]. Similar results were reported also for cryopreserved arterial homografts used to treat patients suffering from aortoiliac or aortobifemoral prosthetic infections [136]. However, the association between graft failure and immunologic injury is still debated. In light of this, pre-clinical studies aimed at validating the quality of cryopreserved grafts before clinical translation appear to be fundamental for controlling and standardizing the preparation methods of these materials in order to increase their biocompatibility.

Together with cryopreservation effect on immunogenicity also the “most adequate decellularization grade” is under controversy. As stated above, to obtain a completely decellularized trachea while preserving tissue ultrastructure/organization and ECM proteins content represents a difficult challenge to face. Because of this, many Authors started to eventually consider the development of partially acellular tracheas [31,33,100,101,102,113,114]. Cells removal from the mucosa/submucosa (respiratory epithelium and glands) decreases trachea antigenicity [137]; whereas cartilage could be identified as an “immune privileged” component [100] due to vessels absence and isolated/masked chondrocytes within a dense collagen-proteoglycans ECM [112]. Despite this encouraging assumption, presence of residual donor cells in the tissue to implant may affect graft safety, as eventual host reactions cannot be excluded. Furtherly, immunosuppressive therapy would be required after surgery, excluding malignancies-suffering patients from this kind of approach.

### 4.3. ECM and Structural Proteins Content Modification

Genetic material removal/reduced immunogenicity while retaining scaffold function is an ambitious goal to pursue [138]. Hence, preservation of the native ECM ultrastructure and composition during cryopreservation and decellularization is highly desirable. CryoT and DecellT grafts were preliminarily compared by H&E for an overall overview on tissue organization and/or evidence of eventual modification/disruptions. Cryopreservation only caused a partial exfoliation of the respiratory epithelium, in accordance with many other studies reported in the literature [24,60,83,97]. In accordance with Nakanishi et al. [83] it is possible that partial maintenance of epithelial cells in tracheal allografts, eventually capable of survival, may be ascribed to cryoprotection itself. Mucosa and submucosa were still identifiable, no damage occurred within the cartilaginous compartment showing well preserved lacunae; however, less DAPI/haematoxylin-stained elements were showed. Nakanishi et al. [8] sustained that cryoprotectants may not penetrate deeply into cartilage leading to chondrocytes degeneration with decreased antigenicity. As regards the adventitia, cellular presence was still detectable, but they were dispersed in a less compact tissue. Despite some expected modifications, overall, CryoT appearance resembled that of NativeT, in accordance with other authors facing tracheal cryopreservation [38,87,93]. Possibly, the adequate freezing rate together with DMSO and albumin presence provided a synergic role in the maintenance of the tissue characteristics, depressing the freezing temperature of water and inhibiting ice formation [139]. Regarding decellularization, the main effects were reported at the respiratory epithelium, appearing as completely denuded. Only the basal membrane was identifiable; this is an intriguing feature, considering that the preservation of this structural element is supposed to facilitate cells’ attachment, viability, and proliferation during repopulation, providing for functional epithelialization following orthotopic transplantation [31,32,140]. Focusing on the cartilaginous compartment, a certain dilation of the lacune was observed as possible consequence of the lyophilization. Lyophilization induces intracellular ice crystal formation, osmotic dehydration, and mechanical forces during rapid freezing with consequent cell membranes disruption, fragmentation of genetic material up to cell lysis. This physical strategy may be associated with ECM modification as well; however, as discussed above, fluids absorption optimization may minimize the amounts of chemical agents required for effective decellularization and their toxic effect [124]. DecellT adventitia, similarly to that of CryoT, appeared less dense but still identifiable. In parallel, H&E data were supported by SEM analysis; the respiratory epithelium modifications, exfoliation in CryoT and denuded in DecellT, were clearly recognizable versus NativeT.

Together with full-thickness tissue organization, the protocol’s impact over ECM proteins was also verified. A fundamental consideration for organ decellularization is minimizing the undesirable alteration and loss of ECM components [126]. Typically, tissues matrix distinguishes fibrous elements (e.g., collagen and elastic fibers) and macromolecules (e.g., proteoglycans) organized in a network [141]. As regards the trachea, its viscoelastic properties convey from its ECM composition of glycosaminoglycans (15–30%), collagen (50–75%), and water (70–80%) [142,143]. Although ionic detergents in tracheal decellularization are very successful, they may affect the natural tissue structure disrupting ECM structure, eliminating growth factors and/or denaturing essential proteins [124,144]; thus, the non-ionic detergent Tergitol^TM^ was preferred. Here, GAGs, collagen (also focusing on collagen type I and type III), elastic fibers were also analysed through the integration of different approaches for a broad description.

Considering GAGs, Sutherland et al. [119], in a comparative study focused on physically devitalized cartilage (freezing at −20 °C, lyophilization, processing in a freezer-mill and frozen at −20 °C) versus decellularized cartilage (physical + enzymatic + chemical method), showed no significant effect after devitalization; conversely, decellularization led to 55% reduction in GAGs content. The same was also highlighted within our study, despite the total GAGs were slightly higher (reduction of 43.6%). GAGs’ role is fundamental in preserving an adequate mechanical behaviour of the tissue, influencing collagen fibrils formation [142]; moreover, they support chondro-induction. However, partial reduction in GAGs content might be beneficial to create a less dense matrix that allows for cell infiltration and migration [119,145]. Masson’s Trichrome staining confirmed collagen maintenance in DecellT and CryoT at the cartilagineous compartment, with a histological appearance resembling that of NativeT. This is essential to assure cartilage integrity [143]. This evidence was also supported by quantification analysis, showing no differences among groups at this level. Differently, a higher content in collagen for DecellT than CryoT and NativeT was calculated at the mucosa/submucosa and adventitia sides. Likely, this descends from removal of cells-associated red-coloured elements that masked the green-intensity within the mucosa/submucosa. Focusing on adventitia, this layer appeared more expanded after decellularization thus resulting in a more consistent area to measure in the photomicrographs. Furtherly, also Sirius Red staining was done, allowing for a specific focus over the mucosa/submucosa/cartilage side and adventitia. Under polarized light, a reduction in collagen type I signal (red-orange colour) was identified for the DecellT group within the mucosa/submucosa compartment versus CryoT and NativeT (these showing the same appearance); here, collagen type III was scantly represented in the whole cohort. An opposite trend was observed within the adventitia, where both collagens were mainly represented in DecellT than in the other groups. Possibly, this may depend on structural modifications following decellularization.

Intriguingly, thanks to advanced optical imaging techniques including multi-photon microscopy, it is possible to describe fibers’ architecture in tissues through SHG, without recurring to any staining/fixation thus integrating the previous analyses [44,45,47,146,147,148]. Typically, collagen type I, II, III and V can produce SHG signals, differently from collagen type IV. Revising the literature, trachea SHG imaging is often limited to cartilage [147]. Ayyalasomayajula and Skallerud [147] provided data on the microstructure of the different tracheal components (mucosa/submucosa, cartilage, adventitia, and trachealis muscle layers), serving as a comparison. Here, together with cartilage, adventitia was also evaluated. Type II collagen was confirmed as the dominant protein of the hyaline cartilage; without any identifiable spatial organization (meshwork-like), it surrounded the lacunae. Hence, in accordance with the description by Ayyalasomayajula and Skallerud [147] on the bovine trachea, collagen in the adventitia was observed to be organized in thick type I collagen bundles emitting an extremely strong and robust SHG signal. These data are in accordance with polarized-light images after Sirius-Red staining.

Together with GAGs and collagen, elastic fibers’ retain after cryopreservation and decellularization was analyzed. Little research on the elastic network in the major airways exists, even though their importance is outstanding providing for trachea ability to increase in length, diameter, being also involved in its elastic recoil [149]. Additionally, as well as collagen, the elastic fibers system has a fundamental role in tissue regeneration and recipient cells in-growth. In the human trachea, elastic fibers are abundantly represented in the submucosa with bundles displaying a longitudinal organization. Circular fibers are also detectable in close contact to the cartilage [149]. This organization resembles that of the pig NativeT and is also identifiable in the CryoT counterpart. Focusing on the DecellT, even if the elastic fibers elements were scant at the mucosa/submucosa layer (which appeared swelled), they were maintained at the interface with cartilage. Differently from CryoT, only elastic fibers remnants were maintained at the adventitia side. Histological evidence was well supported by morphometric analysis data. At the mucosa/submucosa side, NativeT and CryoT were comparable; differently, adventitia was more affected by treatments as highlighted by quantification study.

### 4.4. Compressive Mechanical Properties

Suitable mechanical properties are fundamental in the development of tracheal substitutes, in order to avoid post-surgical occlusion and collapse. Indeed, insufficient stiffness of substitutes and the occurrence of intratracheal stenosis are closely related, being the major reason of failure in tracheal reconstruction. In this framework, the characterization of the compressive mechanical behaviour of the native trachea and potential substitutes is crucial for developing successful surgical solutions [150]. The compressive mechanical properties of NativeT, DecellT, and CryoT were compared following a literature test protocol [151]. Ring-like samples had homogenous sizes, to avoid size- and shape-dependent effects on the compressive behavior. The compressive behaviour of NativeT was consistent with literature data on porcine trachea [116,151]. CryoT did not show any significant difference in stiffness with respect to NativeT, while DecellT had a strong stiffness reduction. This means that DecellT could be occluded up to 50% of compressive deformation with a force per unit of length which is one order of magnitude lower with respect to NativeT. This decrease in the compressive modulus is consistent with the reduction in GAGs content highlighted by histological analysis.

### 4.5. Confirmation of Tracheal Substitutes Cytocompatibility and Biocompatibility

Cryoprotectants exert a fundamental role in guiding, reducing, or preventing ice crystal formation, in turn protecting the biological structures during preservation and guaranteeing ECM integrity after thawing; however, they bear the risk of possible undesired effects over the biological system [152]. Similarly, detergent type has significant effects on the biochemical composition of the decellularized ECM but also on cytocompatibility [141]. To this purpose, a cytotoxicity extract test was conducted prior to verifying biocompatibility through a heterotopic implant. As reported by Sugishita et al. [153], diffusion washing is necessary to significantly reduce the residual cryoprotectants. Similarly, Milian et al. [14] approaching porcine trachea decellularization by SDS, supported osmotic shock steps with distilled water to lessen undesirable effects of detergents. Following this method, nor CryoT neither DecellT showed toxic remnants adsorption/desorption, influencing in a negative manner human bone marrow-derived stromal cells (HM1-SV40) adhesion and proliferation. DecellT showed better outcomes than CryoT; possibly, the presence of a looser matrix (GAGs reduction), favored detergents removal. Moreover, cytocompatibility was assessed also by investigating CryoT and DecellT ability to sustain the adhesion and proliferation the HM1-SV40 cells. SEM analysis confirmed supports repopulation after 3 and 7 days from seeding, thus suggesting the scaffolds attitude to be colonized by cells prior to be implanted and/or in situ after surgical positioning.

To effectively evaluate the quality of a decellularized tissue, subcutaneous implantation constitutes an important step allowing to describe the efficiency of cell-associated proteins removal and local tissue response to eventually adsorbed chemical substances used during decellularization [62,154]. Regarding CryoT, preclinical evaluation allows to predict adverse reactions that may correlate not only with possible cyoprotectant residues but also with donor vital cells. Explants characterization by histology for general appearance (H&E) and ECM characteristics (GAGs, elastic fibers) showed a similar appearance between CryoT and DecellT, after 14 days from surgery; moreover, von Kossa staining excluded presence of any calcification. Hence, immunohistochemistry focused on lympho-monocytic infiltrate evaluation, distinguishing between the mucosa/submucosa-host latissimus dorsi interface and the adventitia-host subcutis interface, respectively. Matrix resorption takes place mainly through phagocytic cells such as mast cells, dendritic cells, and macrophages [155]. According with the analyses performed, a certain immunoreactivity (CD3, F4/80) was detected at both sides for the two experimental groups. It is possible that lymphocytes, exhibiting a certain cytokine secretory activity, triggered the recruitment of monocytes/macrophage, in turn implied in graft degradation and possible remodeling [44,45]. No substantial differences, nor in immune cells infiltration neither in fibrotic capsule formation, were highlighted comparing the CryoT and the DecellT sample groups. SEM explants analysis showed the presence of host cells covering the implant’s surfaces.

Development of an efficient substitute for tracheal reconstruction remains a significant challenge. However, despite intense effort still being needed, the study results confirm that trachea allografts/xenografts may be a compliant option to resort to. Within this promising scenario, large animal studies are a fundamental stage to validate tissue engineered constructs due to sizes resembling that of human anatomy and allowing to face technical challenges, similar to those in clinical practice [117]. In consideration of this, orthotopic implants in an animal model of disease will represent the next step of this comparative work, allowing to analyze CryoT and DecellT ability in overcoming common issues in tracheal reconstruction. Re-epithelialization and vascularization of the grafts are fundamental to achieve functional graft survival.

## 5. Conclusions

Several studies focused on trachea cryopreservation or decellularization; however, according to our knowledge, this is the first investigation providing an in vitro and in vivo comparative analysis of the impact of both methods over tracheal tissue characteristics.

DecellT, developed here, appeared as a non-immunogenic substitute; lyophilization assured for adequate detergents/enzymes penetration, only partly depleting GAGs content; furthermore, Tergitol^TM^ detergent showed favourable results in terms of collagen preservation. In parallel, CryoT maintained native tissue characteristics, while guaranteeing a surprising reduction in genetic material content after freezing and thawing. However, as most cell nuclei were still detectable in whole tissue thickness, immunosuppression would be required in the perspective of an in vivo implant. According to the study evidence, both substitutes, despite being promising, showed strengths and weaknesses; hence, preclinical studies are unavoidable to guide clinicians. It is also possible that, in the future, these two treatments (i.e., decellularization and cryopreservation) will be combined. In fact, cryopreservation may allow for the safe storage of decellularized grafts prior to their use, or storage of native grafts prior to decellularization. Combining these two strategies may also aid in reducing the number of DNase-I-based treatments required for decellularization, and the associated costs of this procedure.

## Figures and Tables

**Table 1:** Size of DecellT, CryoT and NativeT samples.

	d_1_ [mm]	d_2_ [mm]	t [mm]	L [mm]	d_1_ [mm]
**NativeT**	19.6	14.9	2.4	15.9	19.6
	19.8	14.5	2.6	17.3	19.8
	19.7	15.7	2.7	18.5	19.7
	21.6	16.9	2.7	14.7	21.6
**CryoT**	20.0	15.4	1.9	12.6	20
	21.4	16.3	2.7	15.3	21.4
	20.7	16.3	2.3	16.5	20.7
	20.8	15.2	2.1	14.7	20.8
**DecellT**	27.9	13.7	2.4	17.7	27.9
	26.6	11.8	2.8	13.2	26.6
	27.9	13.9	2.8	15.7	27.9

## Data Availability

Not applicable.
